# Environmental impacts and nitrogen-carbon-energy nexus of vegetable production in subtropical plateau lake basins

**DOI:** 10.3389/fpls.2024.1472978

**Published:** 2024-10-22

**Authors:** Yousheng He, Ruifeng Su, Yuan Wang, Shunjin Li, Qi Huang, Xinping Chen, Wei Zhang, Zhi Yao

**Affiliations:** ^1^ College of Resources and Environment, Yunnan Agricultural University, Kunming, China; ^2^ Interdisciplinary Research Center for Agriculture Green Development in Yangtze Lake Basin, Southwest University, Chongqing, China; ^3^ College of Resources and Environment, China Agricultural University, Beijing, China

**Keywords:** subtropical, plateau lake basin, environmental performance, economic benefits, life cycle assessment

## Abstract

Vegetables are important economic crops globally, and their production has approximately doubled over the past 20 years. Globally, vegetables account for 13% of the harvested area but consume 25% of the fertilizer, leading to serious environmental impacts. However, the quantitative evaluation of vegetable production systems in subtropical plateau lake basins and the establishment of optimal management practices to further reduce environmental risks are still lacking. Using the life cycle assessment method, this study quantified the global warming, eutrophication, acidification, and energy depletion potential of vegetable production in a subtropical plateau lake basin in China based on data from 183 farmer surveys. Our results indicated that vegetable production in the study area, the Erhai Lake Basin, was high but came at a high environmental cost, mainly due to low fertilizer efficiency and high nutrient loss. Root vegetables have relatively high environmental costs due to the significant environmental impacts of fertilizer production, transportation, and application. A comprehensive analysis showed that the vegetable production in this region exhibited low economic and net ecosystem economic benefits, with ranges of 7.88–8.91 × 10^3^ and 7.35–8.69 × 10^3^ $ ha^−1^, respectively. Scenario analysis showed that adopting strategies that comprehensively consider soil, crop, and nutrient conditions for vegetable production can reduce environmental costs (with reductions in global warming potential (GWP), eutrophication potential (EP), acidification potential (AP), and energy depletion potential (EDP) by 10.6–28.2%, 65.1–73.5%, 64.5–71.9%, 47.8–70.4%, respectively) compared with the current practices of farmers. This study highlighted the importance of optimizing nutrient management in vegetable production based on farmers’ practices, which can achieve more yield with less environmental impacts and thereby avoid the “trade-off” effect between productivity and environmental sustainability.

## 1 Introduction

Vegetables have become increasingly important with continuously increasing demand (Dijk et al., 2021; Wang et al., 2021). Intensive vegetable production is required to meet this increasing demand; however, it has led to severe environmental damage (Jensen et al., 2024; Lu et al., 2024; Ni et al., 2021). China is the world’s largest vegetable producer and consumer. In 2022, the harvested area and production of vegetables in China accounted for 41% and 53% of the world’s vegetable system, respectively (FAO, 2022). China’s vegetable production system has long employed excessive fertilization to ensure maximum vegetable yields, leading to significant environmental risks and challenges in vegetable production (Kianpoor Kalkhajeh et al., 2021).

The overuse of fertilizers and excessive energy input in vegetable production can directly or indirectly lead to environmental issues such as soil and water pollution, posing serious threats to ecosystem stability and human health, contradicting the sustainable development goal of vegetable production (Kashyap et al., 2023; Martin-Gorriz et al., 2020; Zhang et al., 2013; Zhou et al., 2023). Compared to grain crops, vegetables have a relatively short growing season, and the increasing frequency of input materials (such as fertilizers, pesticides, seeds, diesel, machinery, and agricultural films) during the production process has led to increased environmental emissions (Chen et al., 2021; Romero-Gámez et al., 2014). Zhang et al. (2021) found that nitrogen (N) fertilizers contributed 78.2% of the total greenhouse gas emissions from Chinese vegetable production, making it the largest contributor. In addition, the increasing inputs in vegetable production, particularly the excessive use of fertilizers, leads to higher production costs for vegetables, resulting in decreased economic efficiency for the farmers (Zhang et al., 2024). Governance and restoration of environmental damage also increase economic costs (Lyu et al., 2021; Yao et al., 2021). He et al. (2024) found that, compared to grain crops, vegetable production significantly increased the carbon and N footprints by 2.3–10 and 1.1–2.6 times, respectively, increasing the average environmental damage costs by 2.2 times. Therefore, closely monitoring the environmental and economic benefits of agricultural inputs for vegetable production is crucial.

Lake basins are an important production area for grains and vegetables in China and play a significant role in ensuring the supply of food and vegetables (Sang et al., 2024; Wu et al., 2024). However, agricultural non-point source pollution in plateau lake basins is becoming increasingly serious and cannot be ignored (Huang et al., 2017; Pang et al., 2022; Yu et al., 2020). The agricultural production of the lakes in different regions is relatively different, with a strong regional specificity, resulting in variations in the relevant studies (Grizzetti et al., 2019; Vandamme et al., 2022; Wu et al., 2024). The unique characteristics of tropical/subtropical plateau lake basins can be attributed to their specific geographical and climatic conditions. As a typical subtropical plateau lake basin, the Erhai Lake Basin plays an important role in agricultural production. The surrounding farmland has excellent conditions for vegetable cultivation because of its climatic conditions and abundant water resources (Zhong et al., 2022; Zou et al., 2023). Owing to the lack of reliable agricultural input datasets for vegetable production systems in plateau lake basins, the emission values of vegetable production cannot be quantified, and studies on the environmental impacts of vegetable production systems in these areas are limited.

Here, we conducted a sampling survey of vegetable growers in the Erhai Lake Basin and performed a multi-objective analysis of different types of vegetables using life cycle assessment (LCA) and economic benefit analysis methods. This study aimed to i) quantify the agricultural inputs of the vegetable production system in this region based on household surveys and analyze the environmental impacts (such as global warming, eutrophication, acidification, and energy depletion) and economic benefits [including agricultural economic benefits (EB) and net ecosystem economic benefits (NEEB)] of different vegetable types and ii) assess the environmental impacts and optimization potential of different management practices in vegetable production, consequently identifying effective strategies to improve the sustainability of the vegetable systems.

## 2 Materials and methods

### 2.1 Study area

**Figure 1:**
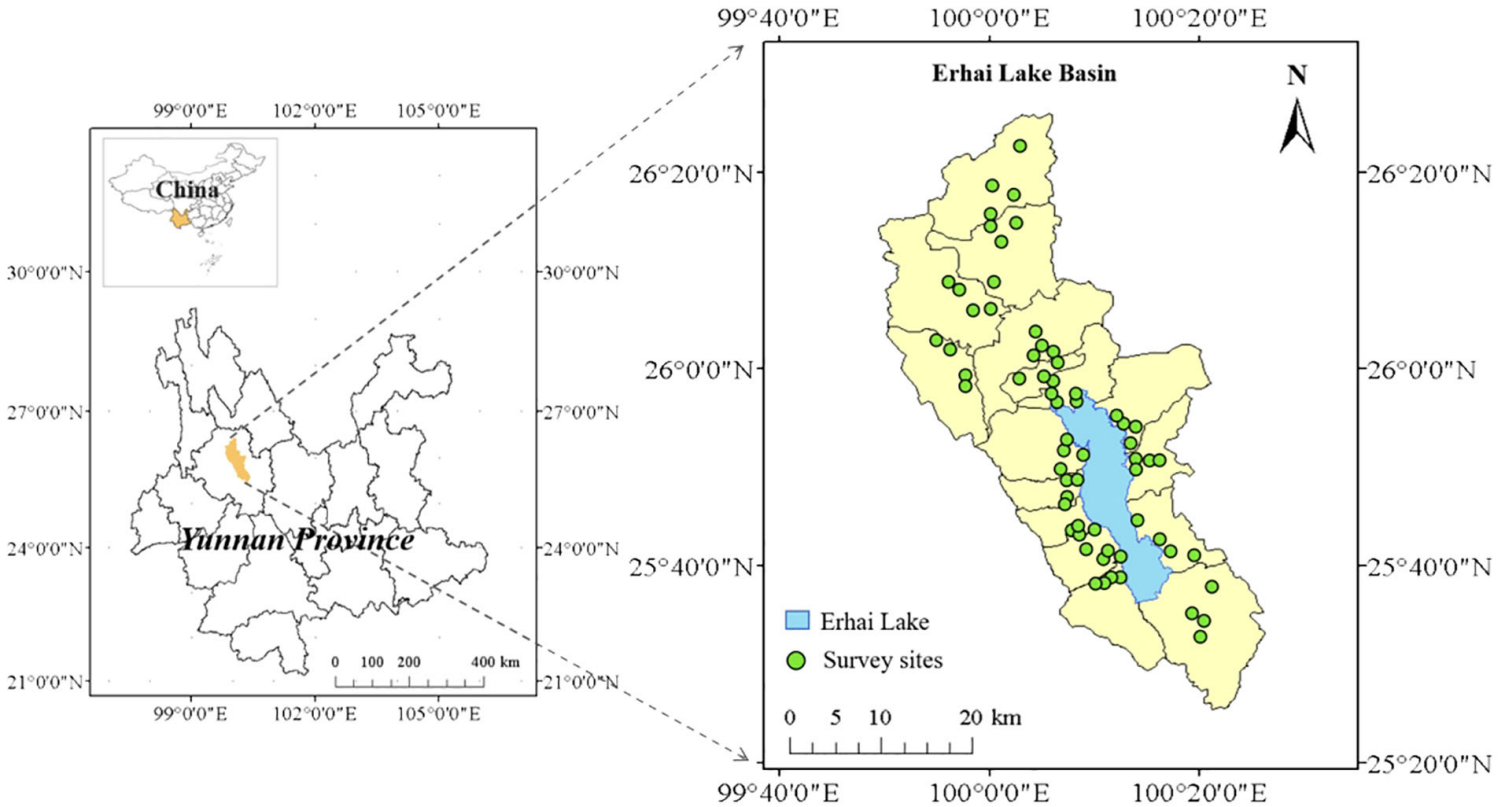
Location of Erhai Lake Basin, a typical low latitude monsoon climate region on China’s subtropical plateau lake basin. The total area of the Erhai Lake Basin area is 2565 km^2^. The cultivated land area is 64 km^2^, of which 24% is planted with vegetables.

This case study was conducted in the Erhai Lake Basin of Yunnan Province, southwestern China (25°25′–26°16′ N, 99°32′–100°27′ E) (**Figure 1**), which has a typical low-latitude plateau subtropical southwest monsoon climate with an annual average temperature of 15.1°C. It receives an annual mean rainfall of 1000–1200 mm, and over 85% of it occurs between May and October, with an annual relative humidity of 66%. The average annual sunlight hours exceed 2000 h, with a sunlight percentage of 56%. Vegetables are the main economic crops in the region because of the superior vegetable production conditions in the region. The multiple-crop index of vegetable fields is high, with no fallow time.

### 2.2 Data collection

The data were obtained through a questionnaire survey conducted in the Erhai Lake Basin in 2022. A random sampling method was used to select the surveyed farmers (Zhou et al., 2023), and face-to-face interviews were conducted with the vegetable growers in the basin to investigate their vegetable production and management practices. There were 16 townships in the Erhai Lake Basin. Four villages were randomly selected from each township, and four farmers were randomly selected from each village for data collection. After excluding uncertain answers, this study ultimately obtained 183 valid data points covering nine types of vegetables in the local region (including asparagus lettuce, green onion, radish, Chinese cabbage, greens, spring onion, cabbage, pepper, and eggplant), which were classified into root, leafy, and fruit vegetables. This survey data on vegetable production included basic information about the farmers (**Supplementary Table 1**), details on single-season vegetable cultivation (including vegetable types, planting area (**Supplementary Table 2**), sowing and harvesting times (**Supplementary Figure 1**), and vegetable yields), and various agricultural inputs for each season (including fertilizers, pesticides, machinery, diesel, plastic films, electricity, and labor).

### 2.3 Estimation of environmental impacts

#### 2.3.1 System boundary and functional units

This study focused on the production of different types of vegetables, and the system boundary covers the cradle (raw material extraction) to the farm gate (vegetable harvesting), mainly including the agricultural materials stage (MS) and arable farming stage (FS). MS primarily focuses on the production and transport of fertilizers (organic and chemical fertilizers), pesticides, fuels, and plastic films. FS is mainly concerned with the application of fertilizers and pesticides and diesel consumption during the use of machinery. To ensure the accuracy of statistical results, the functional units were defined as per metric ton (Mg^−1^) and per hectare (ha^−1^) of vegetables produced. Irrigation water was excluded because of a lack of data that would not affect the main results of this study.

#### 2.3.2 Life cycle inventory analysis

The life cycle inventory includes all resource consumption and emissions related to the functional units (International Organization for Standardization (ISO), 2006a, 2006b). The vegetable production input data used in this study were obtained from field investigations. The production data for different agricultural inputs for each vegetable were weighted and averaged based on the planting area, and the life cycle inventory results are shown in **Table 1** and **Supplementary Table 3**.

**Table 1:** Life cycle inventory for producing per ha vegetables based on farmer-reported data.

Inventory	Unit	Root Vegetables	Leafy Vegetables	Fruit Vegetables
Plastic film	kg ha^-1^	78.2	27.8	55.5
Electricity	kWh ha^-1^	340	458	179
Machinery	h ha^-1^	41.6	90.8	46.4
Diesel	kg ha^-1^	107	156	105
Insecticide	kg ha^-1^	3.55	2.02	0.672
Fungicide	kg ha^-1^	4.41	1.96	0.461
Herbicide	kg ha^-1^	1.59	1.41	1.38
Labor	h ha^-1^	2715	1555	3530

#### 2.3.3 Life cycle impact assessment

This study used LCA to assess the environmental impacts due to vegetable production, including global warming potential (GWP; kg CO_2_-eq per unit^−1^), eutrophication potential (EP; kg PO_4_-eq per unit^−1^), acidification potential (AP; kg SO_2_-eq per unit^−1^), and energy depletion potential (EDP; MJ unit^−1^). Various environmental impacts were calculated using the LCA methods in accordance with ISO standards 14040 and 14044 (ISO, 2006a, 2006b). The calculation process was as follows:


(1) $${\text{EI}}_{\text{j}}=\sum_{\text{i}}^{\text{n}}({\text{PMS}}_{\text{ij}}+{\text{PFS}}_{\text{ij}})\times {\text{Rate}}_{\text{i}}$$

where EI_j_ represents the potential for the j^th^ impact category; j (=1, 2, 3) represents the impact category, including global warming, eutrophication, acidification, and energy depletion; Rate_i_ represents the i^th^ resource use or the emission of vegetable growth, including the application rates of fertilizers, pesticides, diesel, and plastic film; PMS_ij_ represents the emission potential of j impact categories per kilogram of i input produced and transported; and PFS_ij_ represents the emission potential of j impact categories per kilogram of application i. The emission factors of various inputs in the production and transportation processes are listed in **Supplementary Tables 4** and **5**.

### 2.4 Economic benefits analysis

In this study, the input costs of vegetable production were the sum of various agricultural material inputs (including fertilizers, pesticides, machinery, diesel, plastic films, and electricity) and labor costs, and each input was calculated based on the quantity of inputs and related market prices. The total income was calculated based on vegetable production and related prices. The EB and NEEB were calculated as follows:


(2) $$\text{EB}={\text{M}}_{\text{y}}-{\text{AI}}_{\text{cost}}$$

(3) $$\text{NEEB}={\text{M}}_{\text{y}}-{\text{AI}}_{\text{cost}}-{\text{E}}_{\text{cost}}$$

(4) $${\text{E}}_{\text{cost}}={\text{C}}_{\text{GHG}}+{\text{C}}_{\text{eu}}+{\text{C}}_{\text{acid}}$$

where M_y_ represents the income yield from vegetable production. The prices of each vegetable in this study, based on the local market prices, are listed in **Supplementary Table 6**. AI_cost_ represents the cost of agricultural inputs (including the cost of various agricultural material inputs and labor); it was calculated by multiplying the unit price (**Supplementary Table 7**) by the quantity. E_cost_ represents the cost of ecosystem damage. C_GHG_, C_eu_, and C_acid_ represent the GHG emission, water eutrophication damage, and soil acidification damage costs, respectively (Xia and Yan, 2012). Furthermore, according to data from the National Bureau of Statistics, this study referenced an average exchange rate of 6.8974 Chinese Yuan per US dollar in 2020 (Xian et al., 2023). The market price of CO_2_ was 0.0204 $ kg^−1^ (Li et al., 2015). The eutrophication cost of PO_4_ was 0.6086 $ kg^−1^, and the soil acidification cost of SO_2_ was 0.7143 $ kg^−1^ (Xia and Yan, 2012).

### 2.5 Management practices to improve sustainability

Based on the current farming practices (FP) of farmers and the practices of 183 vegetable growers surveyed, this study proposed three agricultural management strategies to improve the sustainability of different types of vegetable production in subtropical plateau lake basins. These three strategies were as follows: (1) S1 (Soil remediation management): In this strategy, to address the soil health issues caused by excessive chicken manure, such as severe soil-borne diseases, declining soil quality, and nutrient surplus, lime nitrogen (30 kg N ha^−1^) was employed, excessive chicken manure was substituted with compost, and the soil was covered with a plastic film (Wang et al., 2020a, 2020b). (2) S2 (Soil remediation and optimized target yield management): This strategy was based on the FP and S1 practices, aiming to improve the efficiency of light energy utilization and yields (Wang et al., 2022) in vegetable production by achieving the top 25% yield of farmers in the survey. Optimized planting density was determined based on the FP (**Supplementary Table 8**). (3) S3 (Integrated soil-crop system management and integrated knowledge and products strategy): This strategy was based on the FP, S1, and S2 strategies and the study by Wang et al. (2021, 2020b, 2020a) to improve vegetable yield and nutrient utilization efficiency while reducing environmental risks. It included using efficient controlled-release fertilizers during nursery stages, selecting locally optimal vegetable varieties, optimizing sowing dates, and adjusting fertilization levels based on the recommended amounts (**Supplementary Table 8**) for different vegetable productions according to soil nutrient availability and target yields. Other agricultural management measures (including planting methods, irrigation management, pest and disease control, and weed management) were consistent with FP. **Supplementary Table 8** shows the target yields, vegetable varieties, planting densities, soil conditioners, types of organic fertilizers, planting dates, and fertilization amounts for different vegetables under each management strategy.

### 2.6 Sensitivity and uncertainty analyses

We conducted sensitivity and uncertainty analyses of the input factors for vegetable production to demonstrate the reliability of this study. The uncertainty in the environmental impacts of vegetable production is influenced by agricultural inputs and emission factors. The coefficient of variation (CV) for agricultural inputs and emission factors was derived from published literature. For agricultural inputs with limited reporting, we assumed that these inputs and emission factors follow a normal distribution with a CV of 30% (Zhang et al., 2021). An uncertainty analysis was performed with 10,000 simulations using a Monte Carlo simulation add-in software (Crystal Ball, 2005) to analyze the sensitivity of the environmental impacts of vegetable production.

### 2.7 Statistical analysis

The vegetable yields, agricultural inputs, environmental impacts, and economic benefits were compared with the least significant difference (LSD) (*P*< 0.05) using one-way analysis of variance (ANOVA). All data analyses were conducted using the SPSS software (version 27.0; Chicago, IL, USA). All figures were plotted using Microsoft Excel 2019 and Origin Pro (Version, 2021; USA).

## 3 Results

### 3.1 Vegetable yields, agricultural inputs, and partial factor productivity of fertilizers

**Figure 2:**
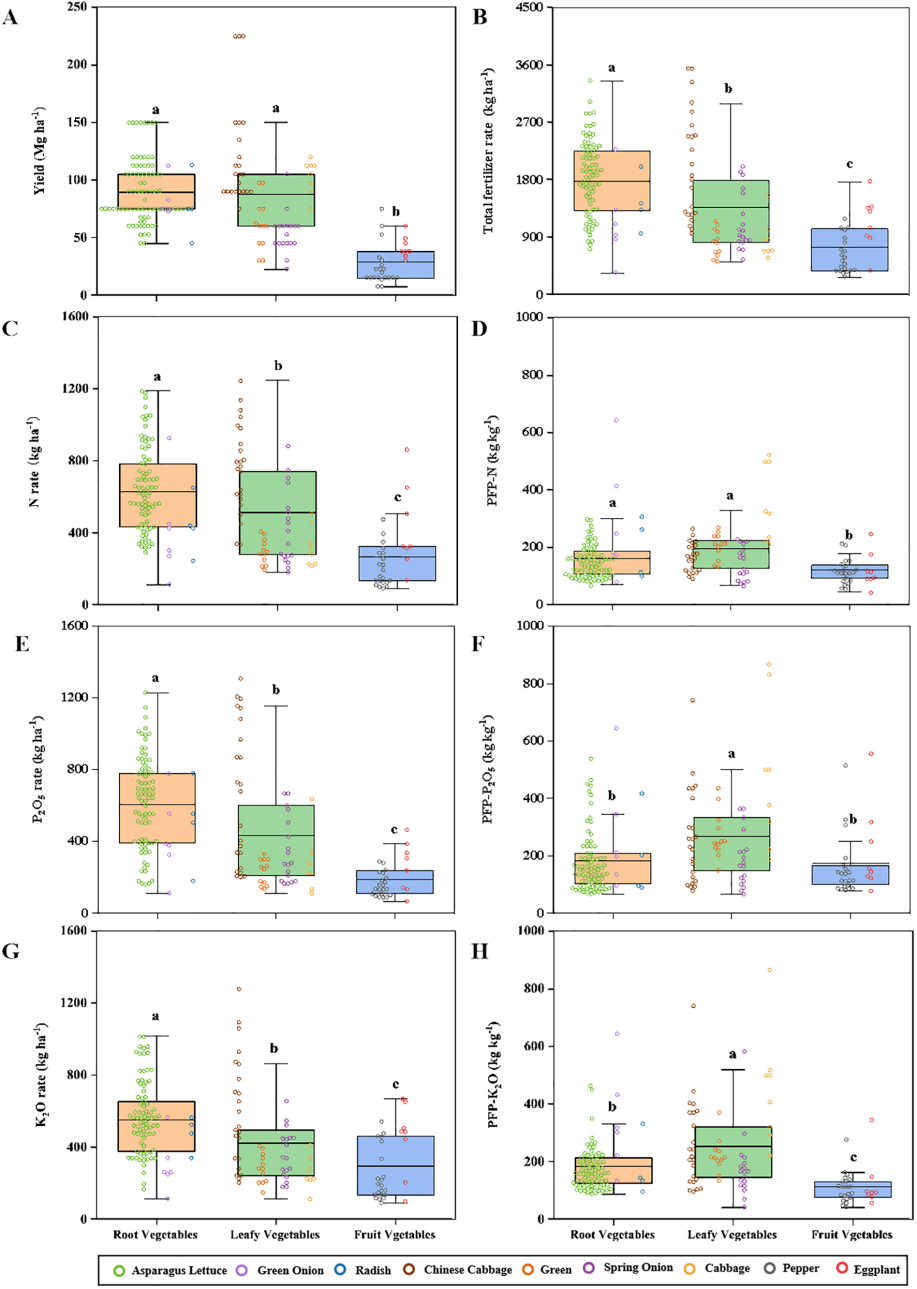
Yield **(A)**, fertilizers inputs **(B, C, E, G)**, and partial factor productivity of fertilizer **(D, F, H)** of different vegetable types in subtropical plateau lake basins. Among them are root (n = 93), leafy (n = 61), and fruit (n = 29) vegetables. Different lowercase letters indicate significant differences among vegetables (*P<* 0.05). The solid black lines inside the box indicate mean values.

The average root, leafy, and fruit vegetable yields in the subtropical plateau lake basin were 89.4, 87.7, and 29.0 Mg ha^−1^ per season (**Figure 2**). The average yield of fruit vegetables was significantly lower than the other two types of vegetables (*p*< 0.05). Chinese cabbage and cabbage had the highest yields at 122 and 103 Mg ha^−1^ per season, respectively. The nutrient application rates (including N, P_2_O_5_, K_2_O, and total fertilizers) for the three vegetable types were significantly different (*p*< 0.05, **Figure 2**). The average total fertilizer application rates for root, leafy, and fruit vegetables were 1787, 1370, and 750 kg ha^−1^ per season (628, 514, and 266 kg N ha^−1^; 608, 434, and 187 kg P_2_O_5_ ha^−1^; and 551, 422, and 297 kg K_2_O ha^−1^ per season), respectively. The agricultural inputs for different vegetables varied significantly, with fruit vegetables having relatively lower input levels but the highest labor consumption time (**Table 1**). The labor requirement for peppers throughout the growth period was 3620 h ha^−1^ per season (**Supplementary Table 3**). Leafy vegetables had the highest partial factor productivity of fertilizer (PFP, **Figure 2**), with PFP-N, PFP-P_2_O_5_, and PFP-K_2_O of 193, 269, and 253 kg kg^−1^ (ranges: 66–524, 67–867, and 41–867 kg kg^−1^), respectively. Cabbage exhibited the highest PFP value.

### 3.2 Environmental impacts of vegetable production

**Figure 3:**
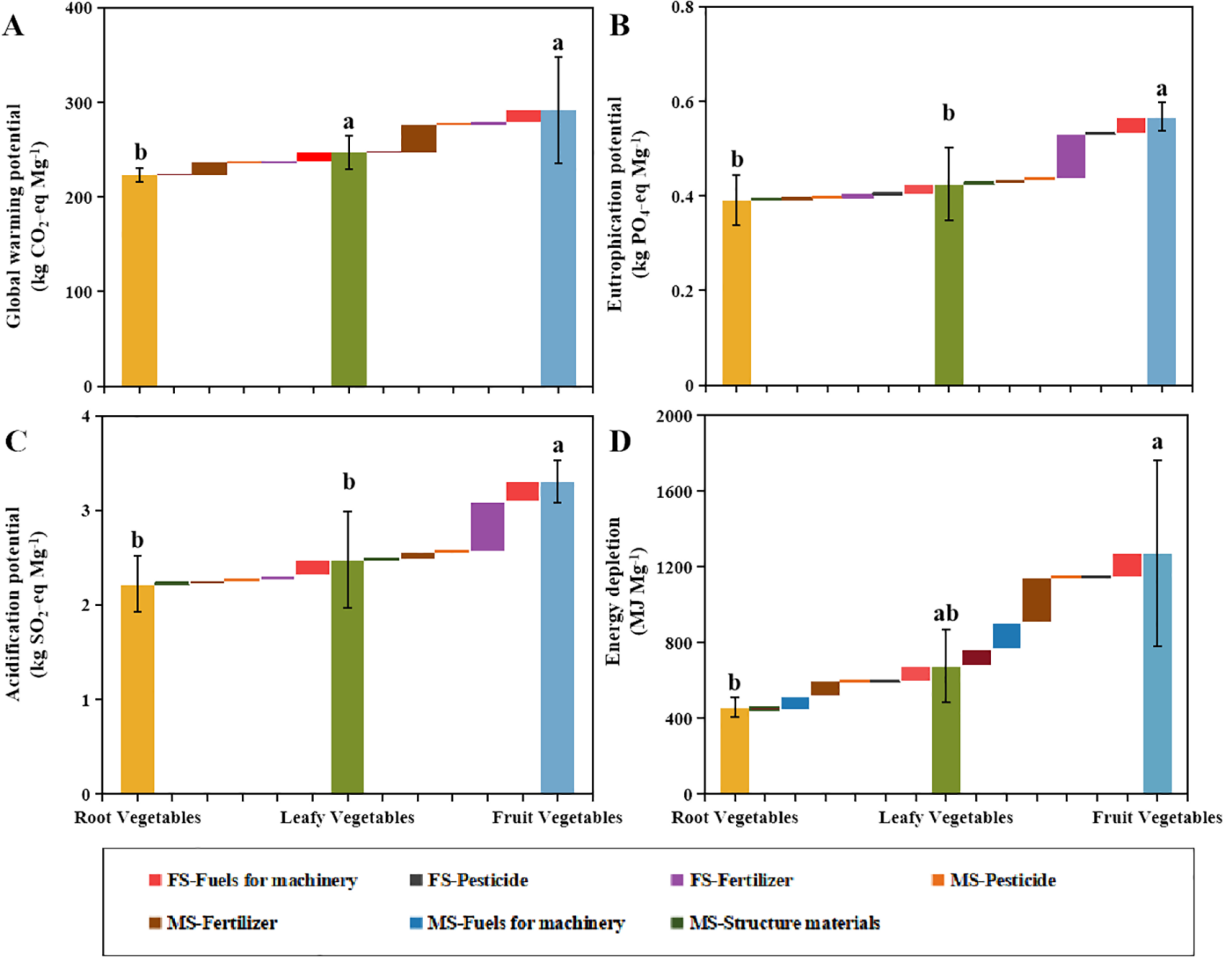
Global warming potential **(A)**, eutrophication potential **(B)**, acidification potential **(C)**, and energy depletion **(D)** of different vegetable production systems. Each environmental impact is influenced by the agricultural materials stage (MS) and the farming stage (FS). MS includes the production and transportation of structural materials, fertilizers, pesticides, and the fuel used for machinery. FS includes the application of fertilizer and pesticides and the use of fuel for machinery. Different lowercase letters indicate significant differences among vegetables (P< 0.05).

The environmental impact of vegetable production was high in the Erhai Plateau Lake Basin, with the impact significantly varying across different vegetable production types (**Figure 3**). Fruit vegetables exerted the greatest impact on the environment (**Figure 3**), with GWP, EP, AP, and EDP values of 292 ± 56 kg CO_2_-eq Mg^−1^, 0.568 ± 0.03 kg PO_4_-eq Mg^−1^, 3.31 ± 0.222 kg SO_2_-eq Mg^−1^, and 1273 ± 493 MJ Mg^−1^, respectively. Moreover, the production, transport, and application of fertilizers, and fuel consumption by machinery of fruit vegetable were higher among all vegetable types (**Figure 3**). Conversely, considering the vegetable production per hectare a functional unit, the environmental impacts of fruit vegetable production were significantly lower than those of the other vegetable types, with GWP, EP, AP, and EDP values of 6787 ± 1522 kg CO_2_-eq ha^−1^, 12.4 ± 3.9 kg PO_4_-eq ha^−1^, 71.8 ± 24.4 kg SO_2_-eq ha^−1^, and 24.9 ± 0.52 GJ ha^−1^ (**Supplementary Figure 2**). Among the three vegetable cultivation methods, fertilizer production, transportation, and application substantially impacted environmental pressure (**Supplementary Figures 2**, **3**). Fertilizer application contributed 69.3–83.2%, 84.4–92.6%, and 80.8–90% to GWP, EP, and AP, respectively. Meanwhile, fertilizer production and transport contributed 50.2–58.3% to EDP (**Supplementary Figure 3**).

### 3.3 Economic benefit analysis of vegetable production

The agricultural input and environmental damage costs of growing root vegetables were relatively high, especially with respect to fertilizer inputs and greenhouse gas emissions (**Table 2**). Furthermore, comparing the yield benefits of different vegetable types revealed that root vegetables performed the best (**Table 2**; **Supplementary Figure 4**), exhibiting 22% and 9.8% higher performance than leafy vegetables and fruit vegetables, respectively. Fruit vegetables exhibited higher economic and ecological benefits, with significantly higher EB (5% and 13.1%) and NEEB (9.6% and 18.2%) than root and leafy vegetables, respectively. According to the agricultural input costs of vegetable production listed in **Supplementary Table 9**, fertilizer, machinery use, and labor costs were the main agricultural expenditures for vegetable production, with labor costs being the highest at 46.2%, 37.5%, and 66.1% for root, leafy, and fruit vegetables, respectively. Greenhouse gas emissions contributed the most to the environmental damage costs, accounting for 72.3%, 75.5%, and 68.3% of the total environmental costs of root, leafy, and fruit vegetables, respectively (**Supplementary Table 10**).

**Table 2:** Comparison of agricultural input costs, environment damage costs, income, economic benefit and ecosystem economic benefit of different types of vegetables in plateau lake basin.

Vegetable types	Agricultural input costs (10^3^ $ ha^-1^)	Environment damage costs (10^3^ $ ha^-1^)	Yield benefit (10^3^ $ ha^-1^)	Economic benefit (10^3^ $ ha^-1^)	Net ecosystem economic benefit (10^3^ $ ha^-1^)
Root vegetables	12.36 ± 2.07 a	0.56 ± 0.15 a	20.84 ± 8.27 a	8.48 ± 3.02 a	7.92 ± 3.97 a
Leafy vegetables	9.21 ± 2.26 b	0.53 ± 0.15 a	17.08 ± 8.30 a	7.88 ± 3.07 a	7.35 ± 3.03 a
Fruit vegetables	10.08± 2.49 b	0.22 ± 0.10 b	18.98 ± 9.49 a	8.91 ± 4.53 a	8.69 ± 4.46 a

Different lowercase letters indicate significantly differences among vegetables (P< 0.05).

### 3.4 Environmental impacts under optimization measures

**Figure 4:**
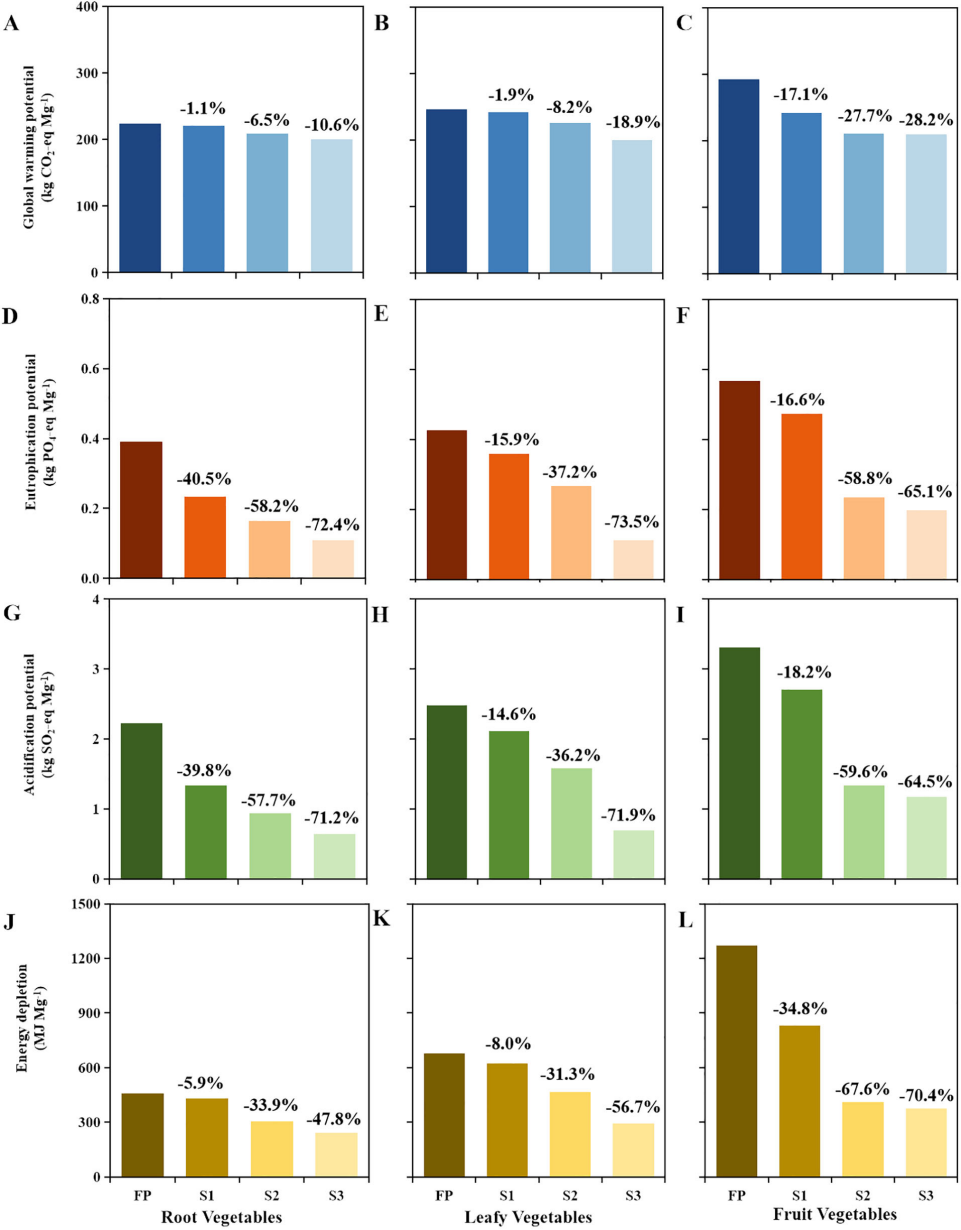
The environmental impacts of different vegetable types under four agricultural management practices in the plateau lake basin. FP is currently a practical treatment for farmers; S1 is soil remediation treatment; S2 is based on achieving the top 25% of household yield under S1 treatment; S3 is integrated soil–crop system management and integrated knowledge and products strategy (ISSM and IKPS). **(A-C)** Global warming potential; **(D-F)** eutrophication potential; **(G-I)** acidification potential; **(J-L)** energy depletion.

Based on scenario simulation, vegetable production using S1, S2, and S3 practices exerted lower environmental impacts than the production using FP. Among them, the S3 management measure exhibited the greatest potential for optimizing environmental impacts (**Figure 4**). The environmental impacts in the S1, S2, and S3 scenarios decreased by 1.1–40.5%, 8.2–67.6%, and 10.6–73.5% compared to the traditional mode (**Figure 4**). In addition, the fruit vegetables under the S3 scenario exhibited a significant optimization potential, with GWP and EDP of 210 kg CO_2_-eq Mg^−1^ and 376 MJ Mg^−1^, reducing by 28.2% and 70.4%, respectively, compared to the fruit vegetables grown via FP.

## 4 Discussion

### 4.1 Yield, nutrient inputs, and economic benefits

All three vegetable types in this study exhibited a high yield (**Figure 2**), generally higher than the weighted average yield of vegetables in the entire Chinese production system (53.8 Mg ha^−1^) and in other regions (Kashyap et al., 2023; Martin-Gorriz et al., 2020; Romero-Gámez et al., 2014; USDA, 2011). Our results showed that leafy vegetables exhibited higher yields among the other vegetable types, which might be attributed to the growth characteristics and moisture content of different crops (Pereira et al., 2021; Zhou et al., 2024). Specifically, the nutritional content varies across different vegetables, with significant differences in nutrient utilization, fruit formation, and the occurrence of diseases and pests (Conversa et al., 2024; Escobar-Bravo et al., 2016). Leafy vegetables can grow rapidly and be harvested relatively quickly, and crop yields can be effectively increased through nutrient management (Corrado et al., 2020), resulting in a higher fertilizer use efficiency. Developing corresponding fertilizer management strategies based on the specific requirements of different vegetable production types is necessary (Zhang et al., 2009) to ensure that vegetables receive a sufficient nutrient supply for high yields. Excessive fertilizer application is a serious concern in vegetable cultivation in the Erhai Lake Basin. Although it leads to high yields, the total fertilizer input for vegetables ranged from 750 to 1787 kg ha^−1^ per season, higher than that of open-field vegetable production in southern China (726 kg ha^−1^ per season) (Zhang et al., 2021). This study observed that the nutrient uptake of vegetables was significantly lower than the fertilizer input, leading to a high nutrient surplus (**Supplementary Table 11**). This phenomenon reflects the issue of excessive fertilization during vegetable production. Meanwhile, the amount of fertilizer used for vegetable production in the Erhai Lake Basin is much higher than that in areas where grain crops are grown in China (Chen et al., 2014; Huang et al., 2017; Kim et al., 2009; Martin-Gorriz et al., 2020). However, the average PFP-N of vegetables in the Erhai Lake Basin is generally lower than that of the entire Chinese vegetable dataset (196 kg kg^−1^) and much lower than some vegetable PFP-N values in other countries and regions (Martínez-Blanco et al., 2011; Mostashari-Rad et al., 2019; Ni et al., 2021; USDA, 2011). Extremely low PFP increases soil N storage levels, exacerbating N leaching, especially under high irrigation conditions (Lv et al., 2019). Therefore, excessive fertilization leads to lower fertilizer efficiency and a higher nutrient surplus in the study area. In particular, excessive N fertilizer application significantly increases N_2_O emissions, NH_3_ volatilization, and N leaching losses (Karlowsky et al., 2021; Kashyap et al., 2023; Romero-Gámez et al., 2014), with summer being more prominent in subtropical plateau monsoon climate zones.

While many vegetable farmers believe that increasing vegetable production can achieve a high economic value, our economic analysis indicates that this may not always be the case. The EB and NEEB in typical vegetable fields in Southeast China (Li et al., 2015), East China (Zhou et al., 2019), and North China (Zhang et al., 2024) are all higher than those in the region. The agricultural inputs in vegetable production in this region were higher than those in other areas, especially with high contributions from fertilizers and labor, at 14.2–31.0% and 37.5–66.1%, respectively. The fragmentation of farmland in the Erhai Lake Basin, and even in the southwestern region, coupled with low mechanization levels, results in a significant labor input requirement throughout the vegetable production lifecycle. Vegetable production in the Erhai Lake Basin is labor-intensive (Kearney et al., 2015), reducing the EB (**Supplementary Table 9**; Zhang et al., 2021). Therefore, mechanized production needs to be developed to increase production efficiency while minimizing the environmental emissions caused by the energy consumption by agricultural machinery (Li and Li, 2022).

### 4.2 Environmental impacts and mitigation potential of vegetable production

Generally, the GWP of the nine vegetables in this study was higher than the average emissions from vegetable production in China (Zhang et al., 2021) and that of common vegetables in other countries (**Table 3**). When considering per hectare of the planting area, the GWPs of Chinese cabbage and asparagus lettuce in the Erhai Lake Basin were 4.3 and 3.2 times that of other vegetables in China, respectively (**Table 3**; Zhang et al., 2021). When considering per ton of vegetable yield, the GWPs of spring onion and pepper in the Erhai Lake Basin were 2.2 and 2.6 times that of other Chinese vegetables, respectively (**Table 3**; Zhang et al., 2021). This finding might be attributed to the high fertilizer input in this study area, far exceeding the nutrient requirements of the vegetables themselves (**Supplementary Table 11**; Ni et al., 2021), as demonstrated in many previous studies (Jensen et al., 2024; Li et al., 2015; Romero-Gámez et al., 2014).

**Table 3:** The environmental impacts of different vegetable production systems in the subtropical plateau lake basin (as determined in this study) and other vegetables and cereal production systems (as determined by a literature search).

Crop	Country /region	Global warming (kg CO_2_-eq unit^-1^)	Eutrophication (kg PO_4_-eq unit^-1^)	Acidification (kg SO_2_-eq unit^-1^)	Energy depletion (GJ/MJ unit^-1^)	References
Unit: per ha						
Asparagus lettuce	Erhai Basin	20019 ± 5645	34.2 ± 11.3	194 ± 64	39.2 ± 12.3	In this study
Green onion	Erhai Basin	17275 ± 2790	22.7 ± 7.9	129 ± 78.6	29.0 ± 11.8	In this study
Radish	Erhai Basin	16398 ± 3237	23.3 ± 8.5	131 ± 46.6	27.8 ± 9.0	In this study
Chinese cabbage	Erhai Basin	26670 ± 6884	39.5 ± 12.2	224 ± 68	42.7 ± 10.5	In this study
Green	Erhai Basin	13447 ± 4297	16.2 ± 3.6	93 ± 19.7	26.4 ± 5.8	In this study
Spring onion	Erhai Basin	13542 ± 4053	23.9 ± 8.2	140 ± 49.5	37.8 ± 9.2	In this study
Cabbage	Erhai Basin	21631 ± 1973	17.5 ± 4.4	100 ± 20.1	28.6 ± 6.8	In this study
Pepper	Erhai Basin	6688 ± 2060	11.9 ± 3.5	69 ± 21.7	24.9 ± 7.0	In this study
Eggplant	Erhai Basin	8839 ± 1919	21.7 ± 8.1	124 ± 43.2	24.2 ± 7.2	In this study
Vegetable	China	6244	–	–	–	Zhang et al., 2021
Peper	China	4060	16.7	87.4	20.3	Wang et al., 2018
Tomato	China	19820	273	452	48.0	He et al., 2016
Escarole	Spain	1318	3.45	9.89	17.0	Romero-Gámez et al., 2014
Lettuce	Spain	1060	3.39	8.48	13.6	Romero-Gámez et al., 2014
Grass-clover	Denmark	1218	4.00	–	3.7	Jensen et al., 2024
Pointed cabbage	Denmark	8366	54.0	–	108	Jensen et al., 2024
Cos lettuce	Denmark	10255	46.0	–	113	Jensen et al., 2024
Onion	Denmark	4988	18.0	–	41	Jensen et al., 2024
Rice	China	10343 ± 1215	–	–	–	Chen et al., 2014
Wheat	China	3707 ± 588	–	–	–	Chen et al., 2014
Maize	China	4436 ± 1140	–	–	–	Chen et al., 2014
Wheat	Sweden	2210	2.18	13.3	13.1	Bernesson et al., 2006
Maize	USA	522-2245	–	–	–	Adviento-Borbe et al., 2007
Unit: per Mg						
Asparagus lettuce	Erhai Basin	223 ± 24	0.394 ± 0.127	2.23 ± 0.71	460 ± 167	In this study
Green onion	Erhai Basin	212 ± 26	0.287 ± 0.093	1.64 ± 0.49	367 ± 102	In this study
Radish	Erhai Basin	225 ± 50	0.340 ± 0.101	1.91 ± 0.63	447 ± 154	In this study
Chinese cabbage	Erhai Basin	221 ± 20.0	0.338 ± 0.103	1.93 ± 0.58	379 ± 128	In this study
Green	Erhai Basin	223 ± 15.6	0.282 ± 0.07	1.63 ± 0.43	468 ± 143	In this study
Spring onion	Erhai Basin	252 ± 22	0.454 ± 0.167	2.65 ± 0.96	739 ± 210	In this study
Cabbage	Erhai Basin	212 ± 18	0.174 ± 0.06	1.00 ± 0.34	290 ± 87	In this study
Pepper	Erhai Basin	296 ± 51	0.570 ± 0.103	3.32 ± 0.79	1305 ± 582	In this study
Eggplant	Erhai Basin	216 ± 27	0.527 ± 0.165	3.01 ± 1.02	608 ± 213	In this study
Vegetable	China	116	–	–	–	Zhang et al., 2021
Peper	China	368	1.52	7.93	1844	Wang et al., 2018
Tomato	China	261	3.59	5.95	628	He et al., 2016
Grass-clover	Denmark	35.0	0.100	–	106	Jensen et al., 2024
Pointed cabbage	Denmark	167	1.10	–	–	Jensen et al., 2024
Cos lettuce	Denmark	256	1.20	–	–	Jensen et al., 2024
Onion	Denmark	139	0.500	–	–	Jensen et al., 2024
Wheat	China	781	6.40	22.4	6324	Liang et al., 2018
Maize	China	443	4.10	13.9	3527	Liang et al., 2018

The environmental impacts include global warming potential, eutrophication potential, acidification potential, and energy depletion potential. The values are expressed per hectare and per metric ton of production.

These results indicated that fertilizer production, transport, and application were the main sources of environmental emissions (**Supplementary Figure 4**). The management practices of local farmers, climate characteristics, soil physicochemical properties, topography, and other external factors can also affect environmental impacts (Huang et al., 2022; Zhong et al., 2021). Machinery and fuel in MS and FS contribute to increased environmental pressure (Jensen et al., 2024; Lovarelli et al., 2018; Romero-Gámez et al., 2014), especially in this region, located in the remote mountainous area of southwestern China (Zhang et al., 2021). Farmlands close to lakes reduce the attenuation of pollutants and exacerbate the risk of non-point source pollution and water pollution in lake basins (Lin et al., 2021).

Compared to grains, vegetables often exhibit higher environmental impacts (**Table 3**), which may be attributed to the shallow root systems and lower nutrient utilization efficiency (Ni et al., 2021). Additionally, to achieve stable yields, farmers frequently increase fertilizer inputs in vegetable production, potentially exacerbating environmental concerns. Simultaneously, significant differences were observed in the environmental risks among the different vegetables in this study. The GWP, EP, AP, and EDP of the fruit vegetables were the lowest when considering per hectare of vegetable yield as a functional unit (**Supplementary Figure 2**). This finding was primarily attributed to the lower inputs throughout the life cycle of fruit vegetables (**Table 1**; **Supplementary Figure 4**). Considering the high environmental risks in the study area, there is significant potential for optimizing vegetable cultivation, with the condition that the optimization measures ensure stable economic profits for farmers.

### 4.3 Optimization measures for soil, crops, and nutrients

**Figure 5:**
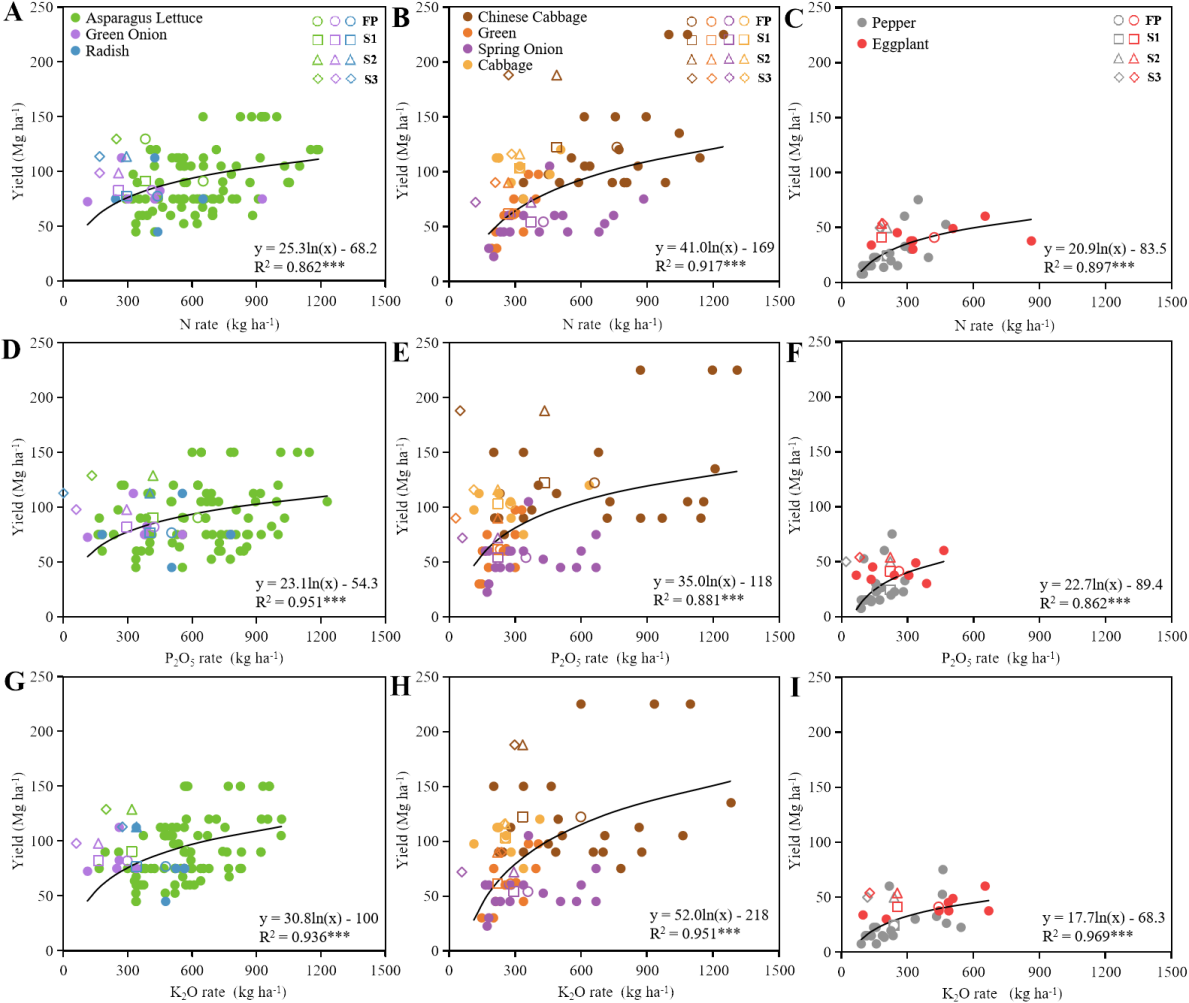
The relationship between fertilizer rate and yield based on surveys of farmers in 2022 for different vegetables. **(A-C)**: N rate; **(D-F)**: P_2_O_5_ rate; **(G-I)**: K_2_O rate. Vegetable types comprised root (n = 93), leafy (n = 61), and fruit (n = 29) vegetables. The lighter dots represent yield under fertilization by surveyed farmers, and the darker shapes (S1, squares; S2, triangles; S3, diamonds) represent yield under fertilization as per the optimization measures. ***P < 0.001.

Optimizing farmland management practices was a key strategy to address the environmental issues posed by the vegetable production system in subtropical plateau lake basins (Wang et al., 2021). Our results indicated that environmental emissions during the fertilization process constitute a significant portion of the environmental impacts (**Supplementary Figure 3**); therefore, optimizing fertilization schemes is an important means to mitigate these environmental impacts. In this study, the relationship between vegetable yield and fertilization levels follows a logarithmic function curve, wherein the vegetable yield initially increases rapidly with rising fertilization levels, followed by a gradual decrease in the rate of increase (**Figure 5**). Some previous studies have reported a logarithmic relationship between fertilizers rates and yields (Topaj and Mirschel, 2018; Kienzler et al., 2011). Accordingly, this study explored certain optimization measures for the vegetable production systems and quantified the mitigation potential of the environmental impacts in the Erhai Lake Basin. Higher yield was achieved with a lower nutrient input through scenarios S2 and S3 (**Figure 5**). The S3 scheme adjusted fertilizer application based on the crop’s nutrient demand and absorption characteristics (**Supplementary Table 11**). It is important to match fertilizer input with nutrient absorption. Best nutrient and crop management strategies are combined with the use of the most effective products to improve root growth and nutrient uptake and to control nutrient losses (Wang et al., 2021). It also increases soil organic matter, improving the soil’s water and nutrient supply capacity (Tei et al., 2020; Thompson et al., 2018). Although soil characteristics, precipitation, topography, and farmers’ practices significantly impact the recommended interventions adversely (Gao et al., 2018), some farmers in the Erhai Lake Basin might achieve a higher yield with a lower nutrient input (**Figure 5**) compared to that achieved using the optimization measures in other regions (especially in Southwest China) (Wang et al., 2020a, 2020b). The recommended interventions not only have strong evidence from field trials but also have reliable empirical support for their effectiveness. The various optimization measures suggested in this study (**Supplementary Table 8**) provide a viable strategy for mitigating environmental impacts in lake basins.

### 4.4 Potential limitations

Although we conducted a comprehensive and detailed analysis of the environmental impacts and economic performance of vegetable cultivation in the Erhai Lake Basin and proposed relevant optimization measures, some potential limitations remain. The Monte Carlo simulations revealed that the 95% uncertainty ranges of GWP, EP, AP, and EDP emissions and NEEB are 177–290 kg CO_2_-eq Mg^−1^, 0.09–0.69 kg PO_4_-eq Mg^−1^, 0.52–3.95 kg SO_2_-eq Mg^−1^, 112–1045 MJ Mg^−1^, and −4147–21705 $ ha^−1^, respectively (**Supplementary Figure 5**). Environmental impacts are primarily influenced by the inputs of various agricultural materials (**Supplementary Figure 6**), with fertilizer exerting the greatest impact. In addition, the yield benefit is a decisive factor affecting NEEB. First, because some farmers could not provide an accurate, specific nutrient composition of their fertilizers, the assessment of this part of the sample data was based on the fertilizer ingredients commonly used locally, especially for organic fertilizers (Kashyap et al., 2023), which might lead to some bias. Second, owing to the lack of localized emission factors, the assessment of environmental emissions was based on the accounting models used by previous studies, which might affect the accuracy of the evaluation results for a specific region. Future research should use local field data or more detailed assessment parameters based on different crops, climates, and management practices. In addition, agricultural market prices fluctuate considerably, and economic benefit analysis is usually based on data from specific periods. Despite these uncertainties and limitations, this study provides a scientific reference for the environmental and economic assessment of vegetable cultivation in this region, contributing to the sustainable development of vegetable production systems in the future. We conducted long-term targeted experiments in the vegetable fields in the region to provide more detailed localized parameters for future studies in this area. This study also offers the emission-reduction strategies for the current green and efficient transformation of vegetable production.

## 5 Conclusions

The environmental performance and ecosystem economic benefits of vegetable production were evaluated using LCA and ecosystem economic analysis based on survey data from farmers in the Erhai Lake Basin. Our results indicated that vegetable yields in this region were higher than the national average and those of other regions in China; however, the large agricultural input led to lower economic performance and high environmental impacts of vegetable production. Excessive fertilization also resulted in low fertilizer efficiency and significant nutrient loss during vegetable production in the study region, resulting in higher environmental costs than those observed in other regions and for other crops. Compared to the current farming practices, integrated agronomic management measures for soil, crops, and nutrients predict that there is significant potential for emission reduction in vegetable planting in the Erhai Lake Basin, reducing GWP, EP, AP, and EDP by 10.6–28.2%, 65.1–73.5%, 64.5–71.9%, and 47.8–70.4%, respectively. Agricultural production must balance economic returns and environmental risks, and our study provided valuable scientific evidence for the sustainable development of vegetable production systems in subtropical plateau lake basins.

## Data Availability

The original contributions presented in the study are included in the article/**Supplementary Material**. Further inquiries can be directed to the corresponding authors.
