# Supplementary material for: Environmental impacts and nitrogen-carbon-energy nexus of vegetable production in subtropical plateau lake basins
Source: Front Plant Sci. 2024 Oct 22;15:1472978. doi: 10.3389/fpls.2024.1472978 (PMC11534706; doi:10.3389/fpls.2024.1472978)

Supplementary Material

- 1. Supplementary Figures


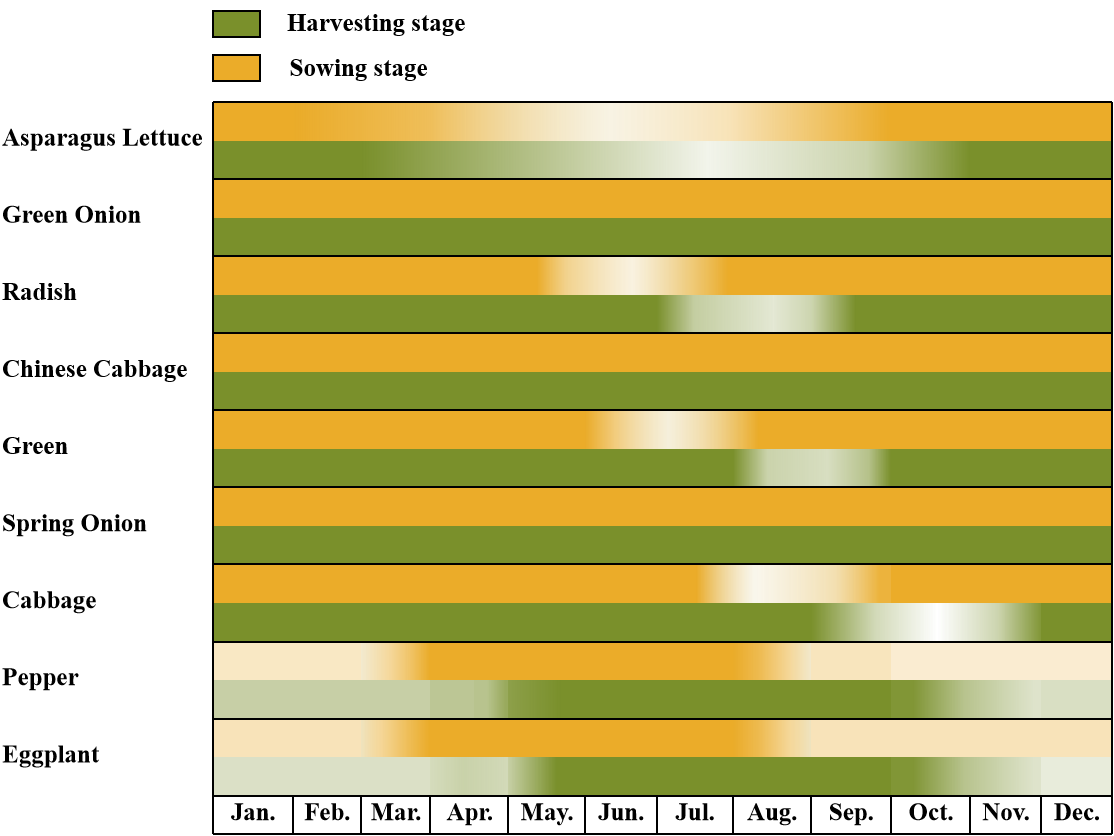


Supplementary Figure 1. The schedule for cultivating 9 types of vegetables by farmers in the Erhai Lake Basin (sowing and harvesting stages).


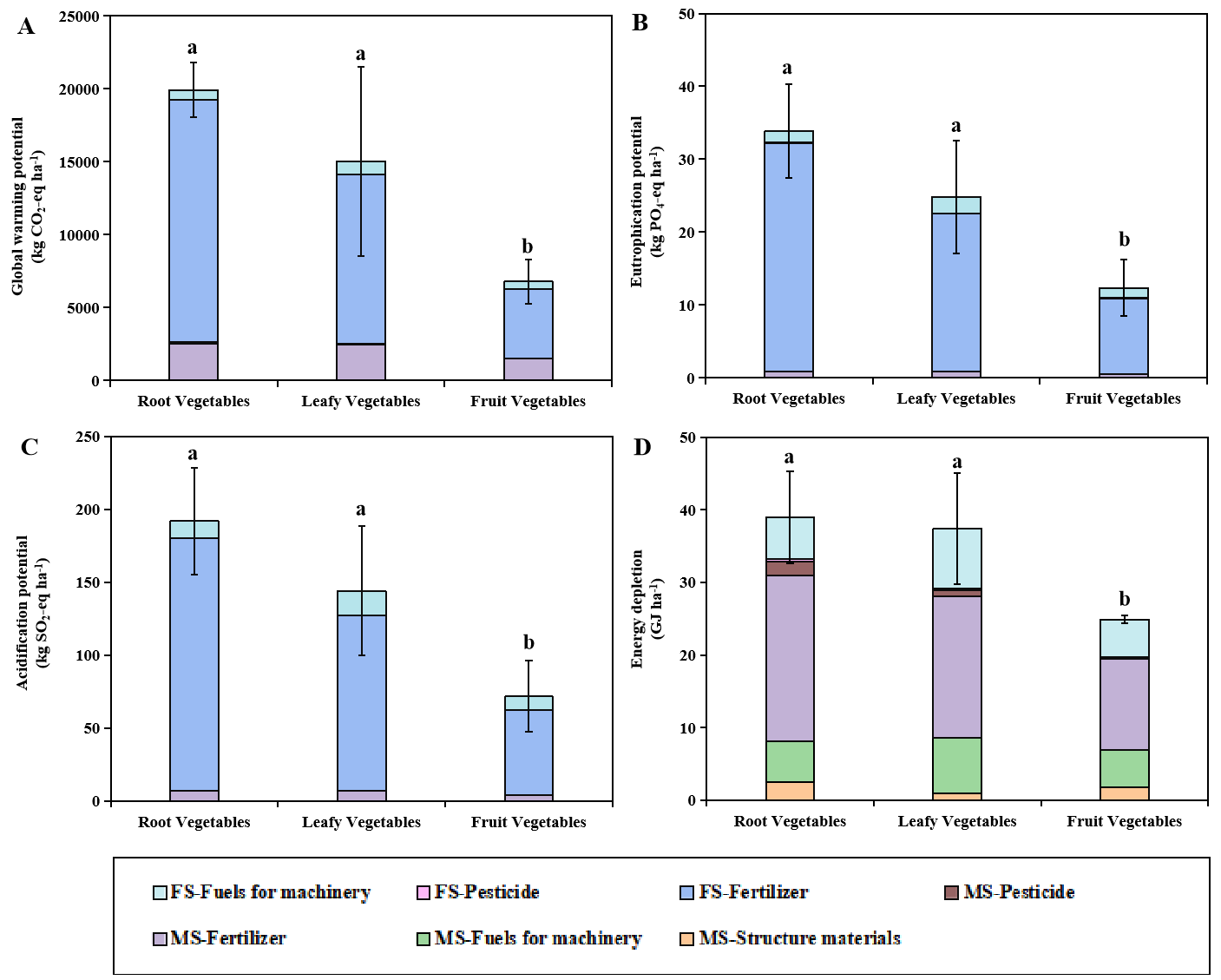
 Supplementary Figure 2. Global warming potential (A), eutrophication potential (B), acidification potential (C), and energy depletion (D) of different vegetable production systems. Each environment impact is influenced by the agricultural materials stage (MS) and the farming stage (FS). MS includes the production and transportation of structural materials, fertilizers, pesticides, and the fuel used for machinery. FS includes the application of fertilizers and pesticides and the use of fuel for machinery.


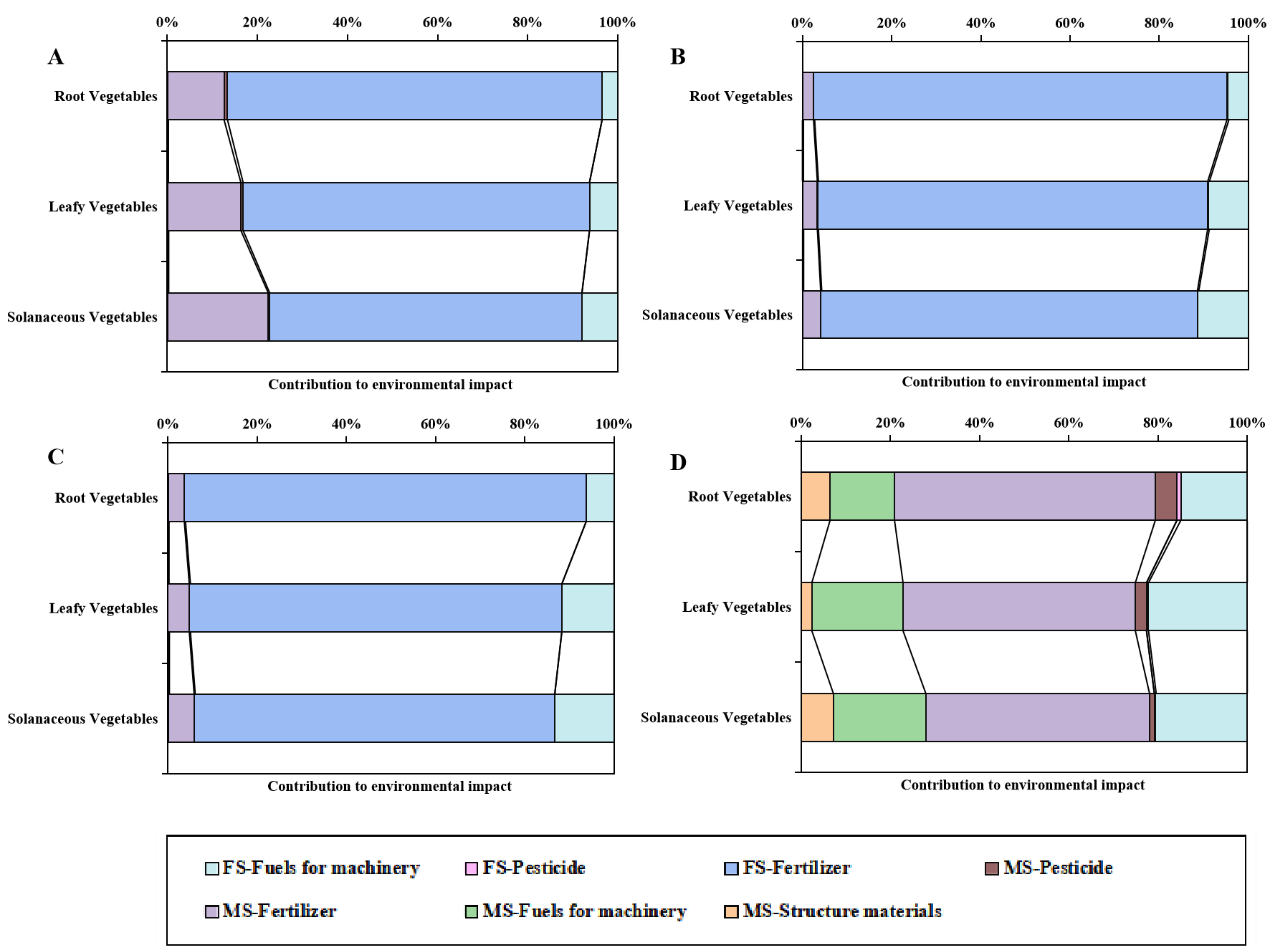
 Supplementary Figure 3. Contributions of inputs to the global warming potential (A), eutrophication potential (B), acidification potential (C), and energy depletion (D) of different vegetable production systems. Each environment impact is influenced by the agricultural materials stage (MS) and the farming stage (FS). MS includes the production and transportation of structural materials, fertilizers, pesticides, and the fuel used for machinery. FS includes the application of fertilizers and pesticides and the use of fuel for machinery.


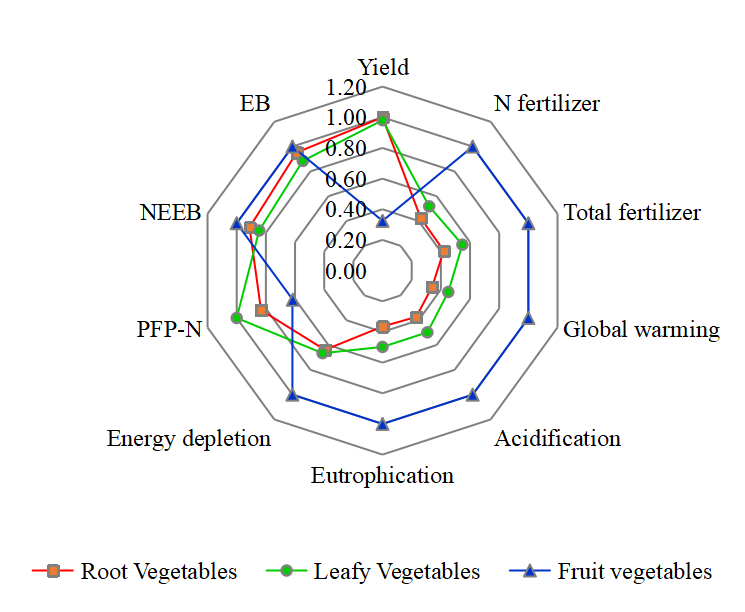
Supplementary Figure 4. Relationships among environmental impacts, fertilizer inputs, vegetable yields, PFP-N, NEEB, and EB among different vegetables production systems on per ton of vegetables production. The environmental impacts include the global warming potential, eutrophication potential, acidification potential, and energy depletion potential.


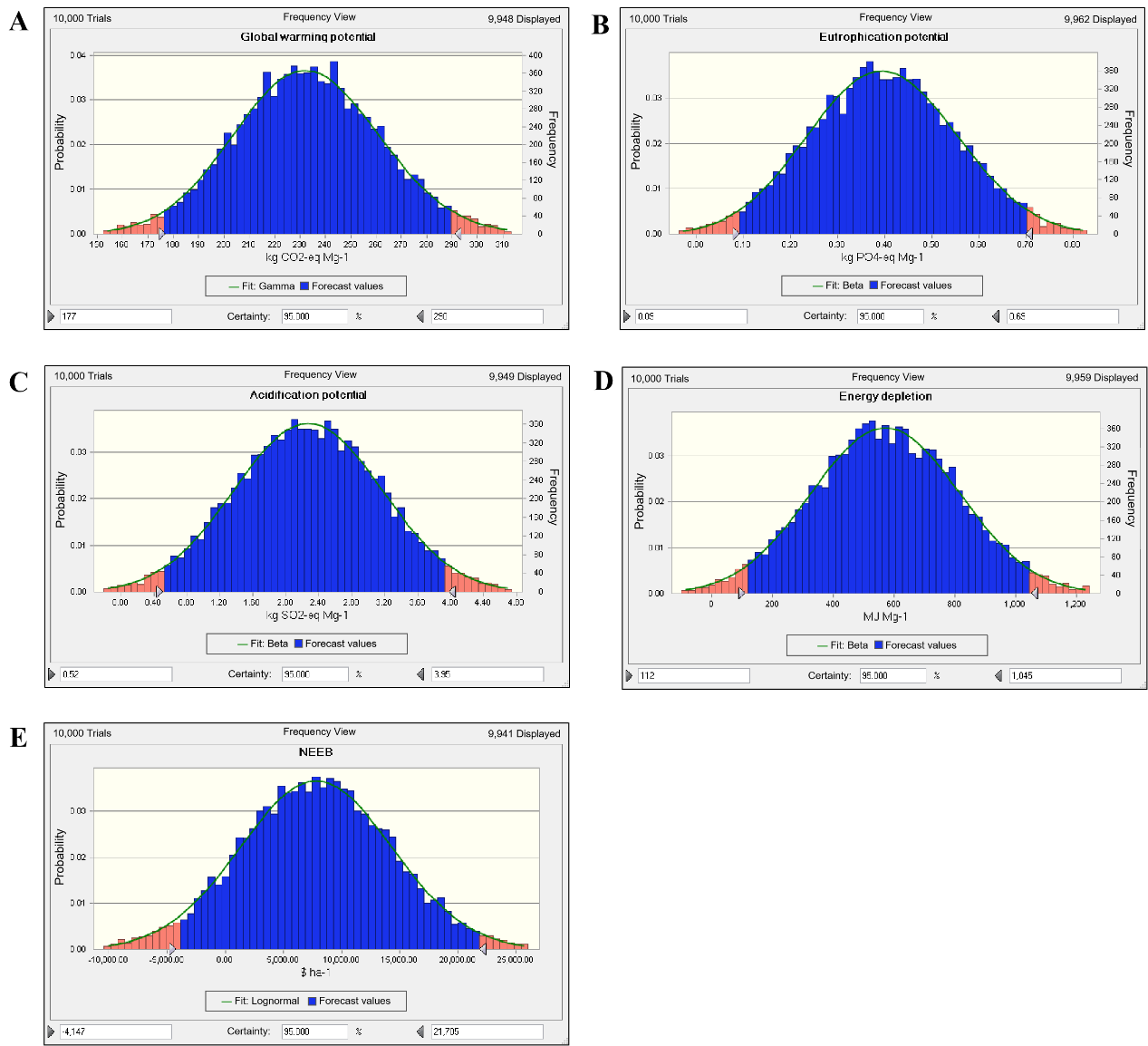


Supplementary Figure 5. Uncertainty analysis of environmental impacts (A, B, C, D) and NEEB (E).


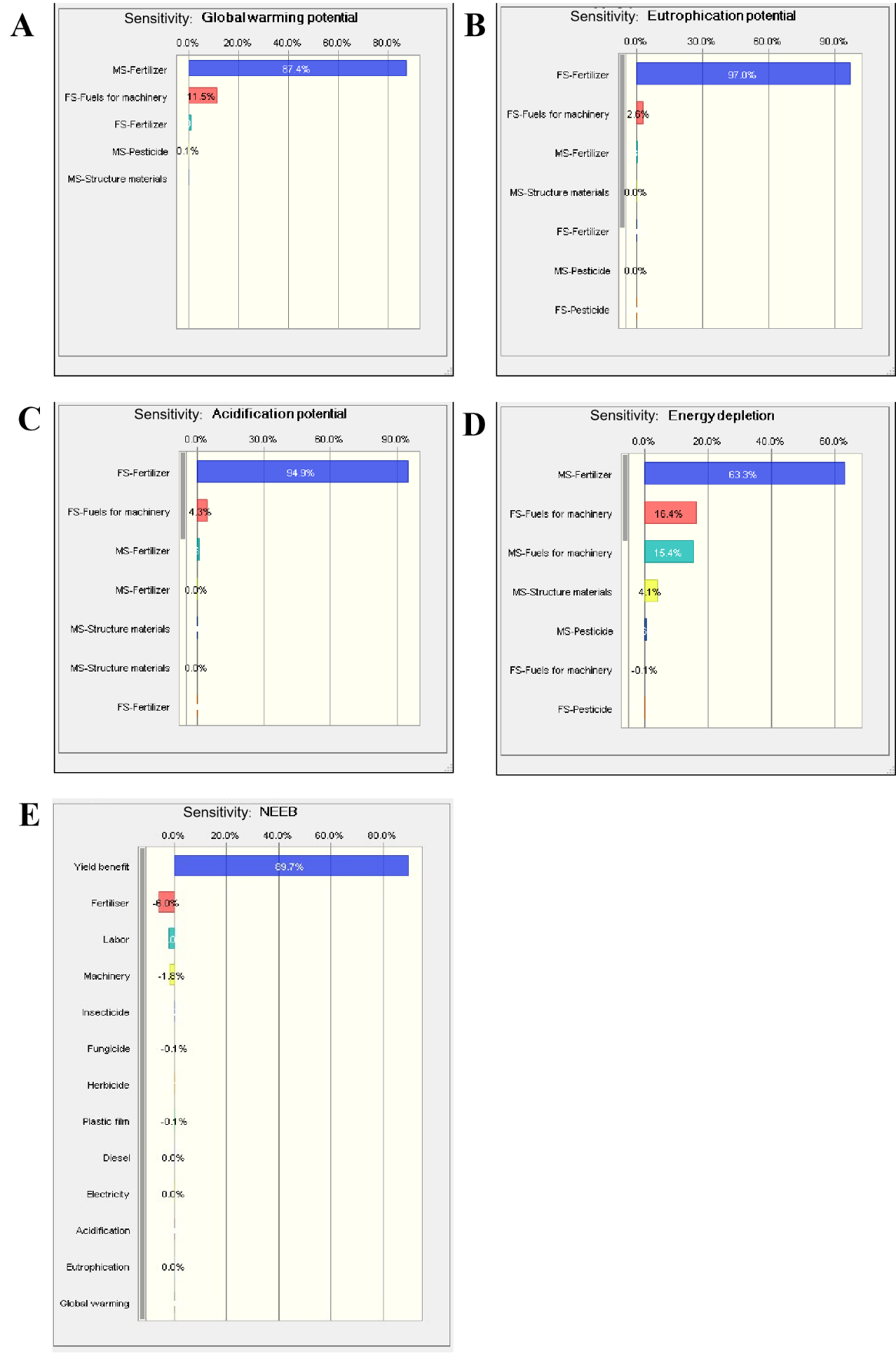


Supplementary Figure 6. Sensitivity analysis for impact factors of environmental impacts and economic benefits.

1.2 Supplementary Tables

Supplementary Table 1. Basic background information of the surveyed farmers.

| Respondent information | Mean | Range |
| --- | --- | --- |
| Age of decision-makers | 52.2±8.9 | 32-75 |
| Years of education (yr) | 8.7±5.0 | 0-17 |
| Annual household income ($ yr^-1^) | 9455±7709 | 1450-46394 |
| Total planting area (ha) | 1.4±2.3 | 0.07-13.3 |
| Number of agricultural labor | 2.3±1.4 | 1-7 |
| Planting experience (yr) | 27.3±13.1 | 2-55 |

Supplementary Table 2. The planting area of the nine vegetables in this study for the year 2022.

| Vegetables | Planting areas (ha^-1^) |
| --- | --- |
| Asparagus Lettuce | 690 |
| Green Onion | 219 |
| Radish | 292 |
| Chinese Cabbage | 374 |
| Green | 372 |
| Spring Onion | 377 |
| Cabbage | 437 |
| Pepper | 2539 |
| Eggplant | 111 |

Supplementary Table 3. Agricultural inputs for the production of each vegetable in this study area.

| Inventory | Unit | Asparagus Lettuce | Green Onion | Radish | Chinese Cabbage | Greens | Spring Onion | Cabbage | Pepper | Eggplant |
| --- | --- | --- | --- | --- | --- | --- | --- | --- | --- | --- |
| Plastic film | kg ha^-1^ | 79.2±29.0 | 0 | 69.4±7.18 | 72.2±29.8 | 30.6±32.8 | 20.8±30.6 | 68.4±6.26 | 50.9±21.3 | 151±29.8 |
| Electricity | kWh ha^-1^ | 342±278 | 360±40.2 | 180±4.08 | 303±202 | 230±107 | 499±439 | 180±2.67 | 178±9.82 | 208±79.6 |
| Machinery | h ha^-1^ | 41.4±27.5 | 80.0±7.75 | 30.0±1.63 | 36.5±21.6 | 52.5±26.0 | 102±59.1 | 30±1.07 | 47.2±20.4 | 30.0±1.07 |
| Diesel | kg ha^-1^ | 107±35.9 | 146±10.6 | 87.0±1.63 | 93.8±20.4 | 105±29.9 | 168±65.9 | 87±1.07 | 106±24.8 | 87.0±1.60 |
| Insecticide | kg ha^-1^ | 3.56±2.98 | 3.59±0.44 | 3.15±0.150 | 3.88±3.42 | 1.97±0.66 | 1.79±1.67 | 2.59±1.30 | 0.552±0.051 | 3.15±0.08 |
| Fungicide | kg ha^-1^ | 4.48±3.05 | 4.20±0.470 | 0.48±0.02 | 3.65±2.05 | 4.05±3.20 | 1.64±1.30 | 2.51±1.16 | 0.46±0.036 | 0.48±0.011 |
| Herbicide | kg ha^-1^ | 1.58±0.334 | 1.90±0.182 | 1.88±0.075 | 1.91±0.184 | 1.91±0.075 | 1.31±0.512 | 1.88±0.106 | 1.36±0.031 | 1.93±0.325 |
| Labor | h ha^-1^ | 2742±314 | 1842±195 | 1539±308 | 1823±236 | 1166±117 | 1539±286 | 1684±74.3 | 3620±869 | 1672±142 |

Supplementary Table 4. Main pollutant emissions factors of agricultural inputs in the vegetables production system at the agricultural materials stage.

| Agricultural  inputs | Unit | Global  warming  (kgCO_2_-eq unit^−1^) | Eutrophication  (kgPO_4_-eq unit^−1^) | Acidification  (kgSO_2_-eq unit^−1^) | Energy  equivalent (MJ unit^−1^) | References |
| --- | --- | --- | --- | --- | --- | --- |
| N | kg | 8.3 | 0.00303 | 0.0252 | 50.5 | Zhang et al., 2013, Cui et al., 2013, Yue et al., 2013 |
| P_2_O_5_ | kg | 0.79 | 0.0000767 | 0.000602 | 5.00 | Zhang et al., 2013, Cui et al., 2013, Yue et al., 2013 |
| K_2_O | kg | 0.55 | 0.0000613 | 0.000482 | 14.7 | Zhang et al., 2013, Cui et al., 2013, Yue et al., 2013 |
| Manure Production | kg | / | / | / | 0.30 | Wang et al., 2018 |
| Pesticides | kg | 19.13 | 0.00194 | 0.0105 | 238 | Clark et al., 2016, Cui et al., 2013, Yue et al., 2013 |
| Plastic film | kg | 0.096 | 0.000103 | 0.00135 | 32.3 | He et al., 2016 |
| Diesel | L | 3.75 | 0.0119 | 0.0658 | 47.8 | Cui et al., 2013, Pishgar-Komleh et al., 2013 |
| Electricity | kWh | 0.75 | 0.00084 | 0.0145 | 12.5 | Yue et al., 2013 |

Supplementary Table 5. Pollutant emission factors during the farming stage in different types of vegetables production.

| Loss pathway | Emission factors | References |
| --- | --- | --- |
| Direct N_2_O emissions | 1.25% of N fertilizer input | Perrin et al., 2014, Wang et al., 2014 |
| Indirect N_2_O emissions | 1% NH_3_ emission +2.5% NO_3_ emission | Perrin et al., 2014, Wang et al., 2018 |
| NH_3_ emissions | 11.1% of N fertilizer input | Ti et al., 2015 |
| NO_3_ emissions | 9.97% of N fertilizer input | Zhao et al., 2010 |
| Phosphorus loss | 0.2% of total P_2_O_5_ fertilizer input | Chen et al., 2011, Wang et al., 2018 |

Supplementary Table 6. The market prices and yield benefits of different vegetables.

| Vegetables | Price ($ Mg^-1^) | Yield benefits ($ ha^-1^) |
| --- | --- | --- |
| Asparagus Lettuce | 217 | 19692 |
| Green Onion | 507 | 41652 |
| Radish | 174 | 13375 |
| Chinese Cabbage | 145 | 17729 |
| Green | 145 | 8907 |
| Spring Onion | 406 | 21989 |
| Cabbage | 159 | 16446 |
| Pepper | 870 | 21201 |
| Eggplant | 319 | 13157 |

Supplementary Table 7. Various unit prices used to calculate agricultural input costs for vegetable production.

| Particulars | Unit | Price ($ unit^-1^)） |
| --- | --- | --- |
| N | kg | 1.02 |
| P_2_O_5_ | kg | 3.99 |
| K_2_O | kg | 1.39 |
| Electricity | kWh | 0.08 |
| Diesel | l | 0.99 |
| Insecticide | kg | 135.48 |
| Fungicide | kg | 120.44 |
| Herbicide | kg | 245.74 |
| Labor | h | 2.17 |
| Plastic film | kg | 2.03 |
| Machinery | h | 26.10 |

Note: The unit prices of electricity, diesel, insecticide, fungicide, and herbicide were referred from Zhou et al. (2023 a), and N, P_2_O_5_, K_2_O, labor, plastic film, and machinery were determined based on the local market price (averaged from 2019-2022). Additionally, the survey showed that machinery was charged by the hour of vegetable production in the Erhai Basin, including purchasing, maintaining, and depreciating agricultural machinery.

Supplementary Table 8. The optimized management information of nine vegetable under different management practices (FP, S1, S2, and S3). FP is the current agricultural practices of farmers in the basin; S1 is the soil remediation management; S2 is the soil remediation and optimized target yield management; S3 is the integrated soil–crop system management and integrated knowledge and products strategy.

|  | Vegetable | Yield  (Mg ha^-1^) | Vegetables variety^1^ | Plant density  (10^3^ plant ha^-1^) | Soil remediation management | Plant date | Fertilizer rate (kg ha^-1^) | | | | References |
| --- | --- | --- | --- | --- | --- | --- | --- | --- | --- | --- | --- |
|  |  |  |  |  |  |  | N | P_2_O_5_ | K_2_O | Total |  |
| FP | Asparagus Lettuce | 91 |  | 113 | No soil conditioner  Chicken manure | Jul. to May of the following year | 652 | 627 | 573 | 1852 |  |
|  | Green Onion | 82 |  | 1425 |  | Annual | 414 | 423 | 298 | 1135 |  |
|  | Radish | 77 |  | 225 |  | Mid Jul. to May of the following year | 440 | 505 | 476 | 1421 |  |
|  | Chinese Cabbage | 122 |  | 63 |  | Annual | 763 | 660 | 601 | 2024 |  |
|  | Green | 61 |  | 336 |  | Aug. to May of the following year | 294 | 232 | 281 | 807 |  |
|  | Spring Onion | 54 |  | 1006 |  | Annual | 428 | 349 | 360 | 1137 |  |
|  | Cabbage | 103 |  | 86 |  | Oct. to mid Jul. of the following year | 321 | 279 | 259 | 859 |  |
|  | Pepper | 24 |  | 65 |  | Late Mar. to mid Aug. | 207 | 159 | 241 | 607 |  |
|  | Eggplant | 41 |  | 52 |  | Late Mar. to mid Aug. | 422 | 260 | 443 | 1125 |  |
| S1 | Asparagus Lettuce | 91 |  | 113 | Lime nitrogen  (30 kg N ha^−1^)  compost fertilizer | Jul. to May of the following year | 383 | 418 | 319 | 1120 |  |
|  | Green Onion | 82 |  | 1425 |  | Annual | 258 | 295 | 163 | 716 |  |
|  | Radish | 77 |  | 225 |  | Mid Jul. to May of the following year | 295 | 403 | 341 | 1039 |  |
|  | Chinese Cabbage | 122 |  | 63 |  | Annual | 489 | 433 | 337 | 1259 |  |
|  | Green | 61 |  | 336 |  | Aug. to May of the following year | 269 | 221 | 222 | 712 |  |
|  | Spring Onion | 54 |  | 1006 |  | Annual | 372 | 221 | 297 | 890 |  |
|  | Cabbage | 103 |  | 86 |  | Oct. to mid Jul. of the following year | 321 | 221 | 259 | 801 |  |
|  | Pepper | 24 |  | 65 |  | Late Mar. to mid Aug. | 207 | 221 | 241 | 669 |  |
|  | Eggplant | 41 |  | 52 |  | Late Mar. to mid Aug. | 182 | 221 | 255 | 659 |  |
| S2 | Asparagus Lettuce | 129 |  | 120 | Lime nitrogen  (30 kg N ha^−1^)  compost fertilizer | Jul. to May of the following year | 383 | 418 | 319 | 1120 |  |
|  | Green Onion | 98 |  | 1350 |  | Annual | 258 | 295 | 163 | 716 |  |
|  | Radish | 113 |  | 225 |  | Mid Jul. to May of the following year | 295 | 403 | 341 | 1039 |  |
|  | Chinese Cabbage | 188 |  | 60 |  | Annual | 489 | 433 | 337 | 1259 |  |
|  | Green | 90 |  | 345 |  | Aug. to May of the following year | 269 | 221 | 222 | 712 |  |
|  | Spring Onion | 72 |  | 1100 |  | Annual | 372 | 221 | 297 | 890 |  |
|  | Cabbage | 116 |  | 90 |  | Oct. to mid Jul. of the following year | 321 | 221 | 259 | 801 |  |
|  | Pepper | 50 |  | 70 |  | Late Mar. to mid Aug. | 207 | 221 | 241 | 669 |  |
|  | Eggplant | 54 |  | 53 |  | Late Mar. to mid Aug. | 182 | 221 | 255 | 659 |  |
| S3 | Asparagus Lettuce | 129 | Hongjianye, Hongxiangfei | 120 | Lime nitrogen  (30 kg N ha^−1^)  compost fertilizer | Oct. to Jan. of the following year | 248 | 133 | 199 | 580 | Chen et al., 2023 |
|  | Green Onion | 98 | Sanyuexiu | 1350 |  | Sep. to Oct. | 170 | 60 | 60 | 290 | Zhang et al., 2009 |
|  | Radish | 113 | Baiyuchun | 225 |  | Mar. to Apr., Aug. to Sep. | 170 | 0 | 275 | 445 | Zhang et al., 2009 |
|  | Chinese Cabbage | 188 | Zhengzao 3, Lubai 8 | 60 |  | Aug. to late Oct. | 270 | 50 | 300 | 620 | Zhang et al., 2009 |
|  | Green | 90 | Lvgan | 345 |  | Late Aug. to early Nov. | 210 | 30 | 240 | 480 | Zhang et al., 2009 |
|  | Spring Onion | 72 | Tieganchun | 1100 |  | Dec. | 120 | 60 | 60 | 240 | Zhang et al., 2009 |
|  | Cabbage | 116 | Xiaotietou | 90 |  | Nov. to Jun. of the following year | 284 | 112 | 255 | 651 | Zhang et al., 2018 |
|  | Pepper | 50 | Honglv 5 | 70 |  | Apr. to May | 175 | 20 | 120 | 315 | Zhang et al., 2009 |
|  | Eggplant | 54 | Qieza 6 | 52.5 |  | Apr. to May | 187 | 82 | 130 | 399 | Ning et al., 2023 |

^1^ Farmers use a variety of cultivars for each type of vegetable, and the specific vegetable varieties in the Erhai Lake Basin under the FP, S1, and S2 strategies are not listed.

Supplementary Table 9. Comparison of input costs of agricultural production for different types of vegetables in subtropical plateau lake basin.

| Vegetable types | Agricultural input costs ($ ha^-1^) | | | | | | | | | Total agricultural costs  ($ ha^-1^) |
| --- | --- | --- | --- | --- | --- | --- | --- | --- | --- | --- |
|  | Fertilizers | plastic film | Electricity | Machinery | Diesel | Insecticide | Fungicide | Herbicide | Labor |  |
| Root Vegetables | 3836 | 150 | 27 | 1132 | 108 | 480 | 516 | 397 | 5713 | 12358 |
| Leafy Vegetables | 2845 | 98 | 26 | 1513 | 116 | 368 | 359 | 424 | 3455 | 9206 |
| Fruit Vegetables | 1432 | 159 | 15 | 1117 | 100 | 172 | 56 | 372 | 6691 | 10115 |

Supplementary Table 10. Comparison of environment damage costs for different types of vegetables in plateau lake basin.

| Vegetable types | Environment damage costs ($ ha^-1^) | | | Total environmental cost ($ ha-1) |
| --- | --- | --- | --- | --- |
|  | Global warming | Eutrophication | Acidification |  |
| Root Vegetables | 402 | 20 | 133 | 555 |
| Leafy Vegetables | 398 | 17 | 112 | 528 |
| Fruit Vegetables | 149 | 9 | 60 | 218 |

Supplementary Table 11. Nutrient input and uptake of different vegetable systems in the study area.

| Vegetable | Yield  (Mg ha^-1^) | Fertilizers input  (kg ha^-1^) | | | | |  | Nutrients uptake  (kg ha^-1^) | | |  | Nutrients surplus  (kg ha^-1^) | | | References |
| --- | --- | --- | --- | --- | --- | --- | --- | --- | --- | --- | --- | --- | --- | --- | --- |
|  |  | N | P_2_O_5_ | | K_2_O | |  | N | P_2_O_5_ | K_2_O |  | N | P_2_O_5_ | K_2_O |  |
| Asparagus Lettuce | 91 | 652 | 627 | | 573 | |  | 212 | 67 | 327 |  | 440 | 560 | 246 | Hong, 2022 |
|  |  |  |  | |  | |  |  | 25 |  |  |  | 602 |  | Yan et al., 2013 |
| Green Onion | 82 | 414 | 423 | | 298 | |  | 117 | 22 | 79 |  | 297 | 401 | 219 | Zhang et al., 2009 |
|  |  |  |  | |  | |  | 157 | 23 | 139 |  | 257 | 400 | 159 | Li et al., 2022 |
|  |  |  |  | |  | |  |  | 24 |  |  |  | 399 |  | Yan et al., 2013 |
| Radish | 77 | 440 | 505 | | 476 | |  | 210 | 43 | 256 |  | 230 | 462 | 220 | Zhang et al., 2009 |
|  |  |  |  | |  | |  | 169 | 31 | 200 |  | 271 | 474 | 276 | Li et al., 2022 |
|  |  |  |  | |  | |  |  | 25 |  |  |  | 480 |  | Yan et al., 2013 |
| Chinese Cabbage | 122 | 763 | 660 | | 601 | |  | 288 | 42 | 155 |  | 475 | 618 | 446 | Zhang et al., 2009 |
|  |  |  |  | |  | |  | 48-258 | 3.2-46 |  |  | 505-715 | 614-657 |  | Zhou et a., 2023 b |
|  |  |  |  | |  | |  | 239 | 50 | 292 |  | 524 | 610 | 309 | Li et al., 2022 |
|  |  |  | |  | |  |  |  | 20 |  |  |  | 640 |  | Yan et al., 2013 |
|  |  |  | |  | |  |  | 253 | 118 | 355 |  | 510 | 542 | 246 | Huang 2017 |
| Green | 61 | 294 | | 232 | | 281 |  | 127 | 26 | 111 |  | 167 | 206 | 170 | Zhang et al., 2019 |
|  |  |  | |  | |  |  |  | 20 |  |  |  | 212 |  | Yan et al., 2013 |
| Spring Onion | 54 | 428 | | 349 | | 360 |  | 40-69 |  |  |  | 359-388 |  |  | Widiana et al., 2020 |
|  |  |  | |  | |  |  |  | 24 |  |  |  | 325 |  | Yan et al., 2013 |
| Cabbage | 103 | 321 | | 279 | | 259 |  | 200 | 80 | 200 |  | 121 | 199 | 59 | Boris et al., 2023 |
|  |  |  | |  | |  |  |  | 20 |  |  |  | 259 |  | Yan et al., 2013 |
|  |  |  | |  | |  |  | 310 | 80 | 274 |  | 11 | 199 | -15 | Huang 2017 |
| Pepper | 24 | 207 | | 159 | | 241 |  | 125 | 11 | 130 |  | 82 | 148 | 111 | Zhang et al., 2009 |
|  |  |  | |  | |  |  | 139 | 26 | 178 |  | 68 | 133 | 63 | Huang et al., 2007 |
|  |  |  | |  | |  |  |  | 48 |  |  |  | 111 |  | Yan et al., 2013 |
|  |  |  | |  | |  |  | 56 | 18 | 86 |  | 151 | 141 | 155 | Huang 2017 |
| Eggplant | 41 | 422 | | 260 | | 443 |  | 147 | 72 | 262 |  | 275 | 188 | 181 | Hong, 2022 |
|  |  |  | |  | |  |  | 127 | 29 | 201 |  | 295 | 231 | 242 | Huang et al., 2017 |
|  |  |  | |  | |  |  |  | 48 |  |  |  | 212 |  | Yan et al., 2013 |

References

Boris, A., Cabilovski, R., Vojnović, Đ., and Ilin, Ž. (2023) Effect of Mulching on Nutrient Uptake and Efficiency of Fertilizers in Mid-early Cabbage Production. *Acta Sci. Pol. Hortorum Cultus* 2269–80. doi: 10.24326/asphc.2023.4813

Chen, X.P., Cui, Z.L., Vitousek, P.M., Cassman, K.G., Matson, P.A., Bai, J.S., Meng, Q.F., et al. (2011). Integrated soil-crop system management for food security. *Proc. Natl. Acad. Sci. USA* 108, 6399–6404. doi: 10.1073/pnas.1101419108

Chen, J. F., Yin, M., Chen, H., Wang, Z. Y., Wang, W., and Fu, L. B. (2023). Study on Fertilization System of Nutrient Critical Value for High Yield of Asparagus Lettuce in Yunnan. *Chinese Agricultural Science Bulletin*, 39, 89-93 (In Chinese)

Clark, S., Khoshnevisan, B., and Sefeedpari, P. (2016). Energy efficiency and greenhouse gas emissions during transition to organic and reduced-input practices: Student farm case study. *Ecol. Eng.* 88, 186–194, doi: 10.1016/j.ecoleng.2015.12.036

Cui, Z., Yue, S., Wang, G., Zhang, F., and Chen, X. (2013). In-Season Root-Zone N Management for Mitigating Greenhouse Gas Emission and Reactive N Losses in Intensive Wheat Production. *Environ. Sci. Technol.* 47, 6015–6022, doi: 10.1021/es4003026

He, X., Qiao, Y., Liu, Y., Dendler, L., Yin, C., and Martin, F. (2016). Environmental impact assessment of organic and conventional tomato production in urban greenhouses of Beijing city, China. *J. Cleaner Prod.* 134, 251–258, doi: 10.1016/j.jclepro.2015.12.004

Hong, J. (2022). Study on Nutrient Absorption and Techniques for High Quality and Efficient Fertilization of Three Main Vegetable Varieties in Wuhan. Ph.D. dissertation. Huazhong Agricultural University, Hubei

Huang, H.G., Zhang, X.Z., Li, T.X., and Yu, H.Y. (2007) Nutrient balance and its environmental risks in typical greenhouse system. *J. Agro-Environ. Sci.* 676-682. doi: 10.3321/j.issn:1672-2043.2007.02.052

Huang, S.W., Tang, J.W., Li, C.H., Zhang, H.Z., and Yuan, S. (2017). Reducing potential of chemical fertilizers and scientific fertilization countermeasure in vegetable production in China. *J. Plant Nutr. Fertil.* 1480-1493. doi: 10.11674/zwyf.17366

Li，S.T., Ai，C., He，P., Zhang，J.J., Cui，R.Z., Wei，J.L., Chuan，L.M. et al. (2022). Characteristics of major Chinese vegetables in nutrient uptake and requirement. *China Veg.* 41-48. doi: 10.19928/j.cnki.1000-6346.2022.2001

Ning, L. Y. R., Qiu, S. J., Xv, X. P., Ding, W. C., Zhao, S. C., He, P., and Zhou, W. (2023). Intelligent fertilizer recommendation method for potato based on yield response and agronomic efficiency. *Journal of Plant Nutrition and Fertilizers*, 29(12), 2272–2281 (In Chinese)

Perrin, A., Basset-Mens, C., and Gabrielle, B. (2014). Life cycle assessment of vegetable products: a review focusing on cropping systems diversity and the estimation of field emissions. *Int. J. Life Cycle Assess.* 19 No. 6, 1247–1263, doi: 10.1007/s11367-014-0724-3

Pishgar-Komleh, S.H., Omid, M., and Heidari, M.D. (2013). On the study of energy use and GHG (greenhouse gas) emissions in greenhouse cucumber production in Yazd province. *Energy* 59, 63–71, doi: 10.1016/j.energy.2013.07.037

Ti, C., Luo, Y., and Yan, X. (2015). Characteristics of nitrogen balance in open-air and greenhouse vegetable cropping systems of China. *Environ. Sci. Pollut. R.* 22, 18508–18518, doi: 10.1007/s11356-015-5277-x

Wang, C., Li, X., Gong, T., and Zhang, H. (2014). Life cycle assessment of wheat-maize rotation system emphasizing high crop yield and high resource use efficiency in Quzhou County. *J. Cleaner Prod.* 68, 56–63, doi: 10.1016/j.jclepro.2014.01.018

Wang, X., Zou, C., Zhang, Y., Shi, X., Liu, J., Fan, S., Liu, Y., *et al.* (2018). Environmental impacts of pepper (*Capsicum annuum* L) production affected by nutrient management: A case study in southwest China. *J. Cleaner Prod.* 171, 934–943, doi: 10.1016/j.jclepro.2017.09.258

Widiana, S., Yuniarti, A., Sofyan, E., and Sara, D.S. (2020) The Effect of NPK Fertilizer on N Total, N-Uptake, and Shallot Yield (Allium ascalonicum L.) on Inceptisols Jatinangor. *Am. J. Appl. Chem.* 8, 152. doi: 10.11648/J.AJAC.20200806.14

Yan, Z., Liu, P., Li, Y., Ma, L., Alva, A., Dou, Z., Chen, Q. et al. (2013) Phosphorus in China’s Intensive Vegetable Production Systems: Overfertilization, Soil Enrichment, and Environmental Implications. *J. Environ. Qual.* 42 (4), 982–989. doi: 10.2134/jeq2012.0463

Yue, S.C., (2013). Optimum Nitrogen Management for High-yielding Wheat and Maize Cropping System. China Agricultural University Press, Beijing, p. 80

Zhang, F. S., Chen, X. P., and Chen, Q. (2009). *Fertilization Guidelines for Major Crops in China*. China Agricultural University Press. (In Chinese)

Zhang, W.F., Dou, Z.X., He, P., Ju, X.T., Powlson, D., Chadwick, D., Norse, D., *et al.* (2013). New technologies reduce greenhouse gas emissions from nitrogenous fertilizer in China. *Proc Natl Acad Sci U S A* 110, 8375–8380, doi: 10.1073/pnas.1210447110

Zhang, M.Q., Li, J., Zhang, Z.D., Yao, B.Q., Xv, W.J., Shen, J.Q., (2018). Agronomic Effects of Recommended NPK Fertilization on Vegetable Farming. Fujian Journal of Agricultural Sciences. 33(10), 1023-1029 (In Chinese)

Zhang, Y., Xie, D., Ni, J., and Zeng, X. (2019) Optimizing phosphate fertilizer application to reduce nutrient loss in a mustard (Brassica juncea var. tumida)-maize (Zea mays L.) rotation system in Three Gorges Reservoir area. *Soil Tillage Res.* 190, 78–85. doi: 10.1016/j.still.2019.03.001

Zhao, C., Hu, C., Huang, W., Sun, X., Tan, Q., and Di, H. (2010). A lysimeter study of nitrate leaching and optimum nitrogen application rates for intensively irrigated vegetable production systems in Central China. *J. Soil Sediment.* 10, 9–17, doi: 10.1007/s11368-009-0063-3

Zhou, Y., Ji, Y., Zhang, M., Xu, Y., Li, Z., Tu, D., and Wu, W. (2023 a). Exploring a sustainable rice-cropping system to balance grain yield, environmental footprint and economic benefits in the middle and lower reaches of the Yangtze River in China. J. Cleaner Prod. 404, 136988. doi: 10.1016/j.jclepro.2023.136988

Zhou, Y., Manu, M.K., Li, D., Johnravindar, D., Selvam, A., Varjani, S., and Wong, J. (2023 b) Effect of Chinese medicinal herbal residues compost on tomato and Chinese cabbage plants: Assessment on phytopathogenic effect and nutrients uptake. *Environ. Res.* 216, 114747. doi: 10.1016/j.envres.2022.114747

Language editing services certificate


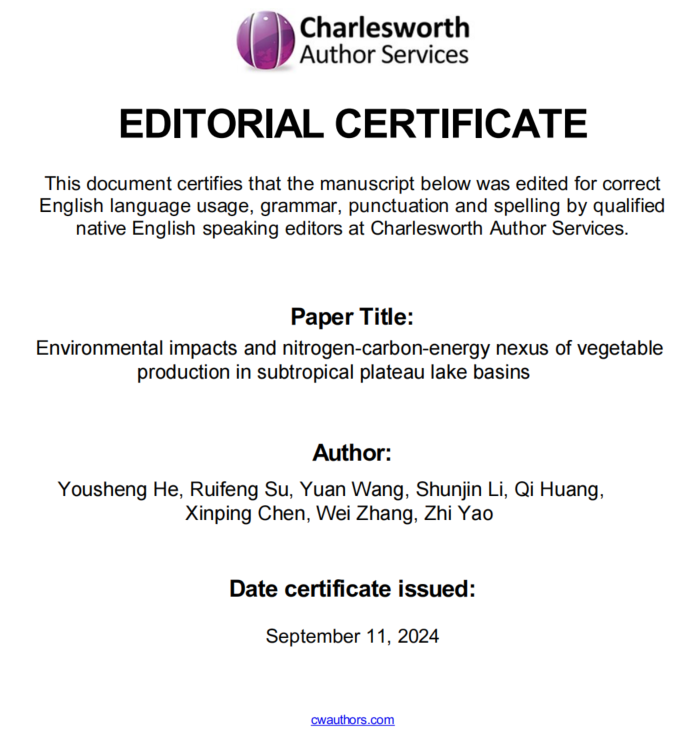


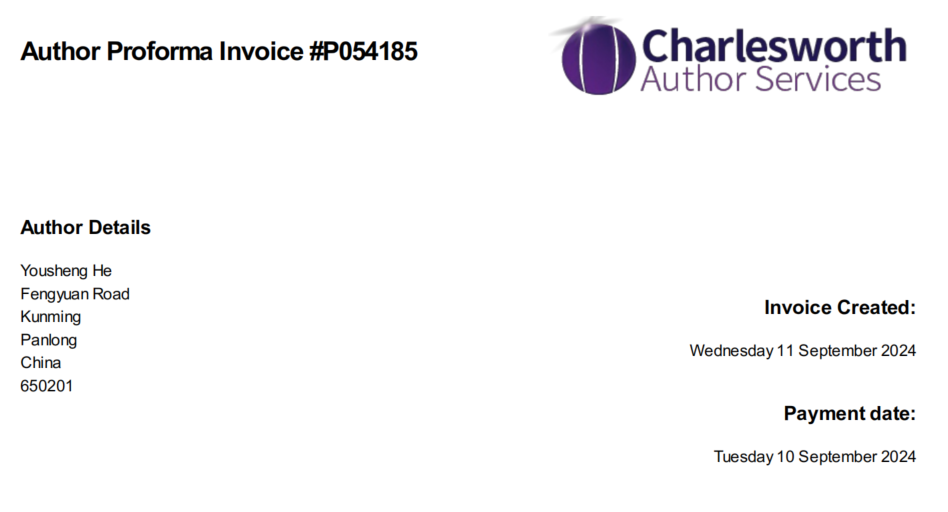

Supplement: Supplementary file 1 [file DataSheet1.docx]
